# Supramolecular Assembly of End-Functionalized Polystyrene and Polydimethylsiloxane with Hybrid Nanostructures

**DOI:** 10.1021/acs.macromol.5c03429

**Published:** 2026-03-17

**Authors:** Jingchao Qin, Whitney S. Loo

**Affiliations:** Department of Chemical and Biological Engineering, 5228 University of Wisconsin-Madison, Madison, Wisconsin 53706, United States

## Abstract

Blends of oppositely charged polymers are capable of self-assembling into well-ordered nanostructures via supramolecular self-assembly. A series of monosulfonated-terminated polystyrene (PS) and monopiperidine–terminated poly­(dimethylsiloxane) (PDMS) polymers with different molecular weights were synthesized and blended to investigate their phase behavior. A new synthetic route for piperidine-terminated PDMS with low dispersity and high end-group fidelity was developed based on a functionalized initiator accessible from commercially available reagents and compatible with anionic ring-opening polymerization. The resulting nanostructure of the blends was measured via small-angle X-ray scattering (SAXS) performed across a range of temperatures and revealed disordered structures in blends prepared with low molecular weight polymers. A model derived by Tanaka using the random phase approximation (RPA) was applied to extract the heteroassociation fraction (*z*) and effective interaction parameter (χ_eff_), and a quantitatively similar value of *z* was observed across all blends with disordered morphologies. High molecular weight blends exhibited a variety of hybrid ordered nanostructures, and by precisely tuning the mixing ratio, we were able to induce phase transitions and achieve a variety of ordered nanostructures with long-range order. A molecular-scale mechanism to balance the electrostatic strength of end group association and segregation strength between the constituent polymers is proposed to rationalize the observed phase behavior.

## Graphical abstract

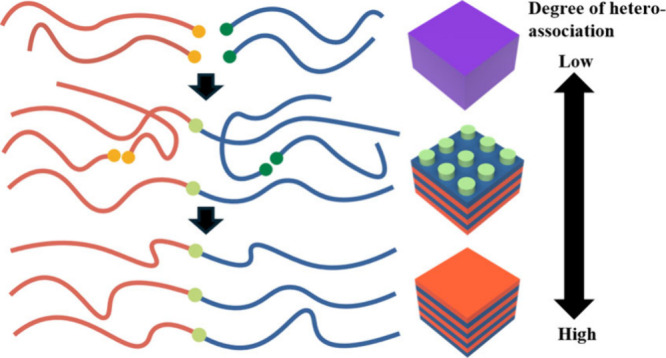

## Introduction

Self-assembly of block copolymers (BCPs) has been extensively studied over the past several decades.^1–8^ By tuning key parameters such as the volume fraction, degree of polymerization (*N*), and the Flory–Huggins interaction parameter (χ), BCPs can self-assemble into a wide range of ordered morphologies—including spheres, cylinders, bicontinuous gyroids, and lamellae, resulting in distinct mechanical, optical, and transport properties.^2,3,9–11^ The driving force for microphase separation is the segregation strength, defined as χ*N*, where *N* represents the total chain length and χ quantifies the enthalpic interaction between chemically distinct monomer subunits. Beyond χ*N*, additional interactions such as hydrogen bonding and ionic interactions can also serve as powerful driving forces for polymer self-assembly. Supramolecular assembly, which is guided by noncovalent interactions, exists widely in nature and plays a vital role in biological systems.^12–16^ In the field of synthetic polymer materials, introducing ionic interactions, which are much stronger than hydrogen-bonding interactions, can drive the formation of well-defined, long-range ordered nanostructures.^17–19^ A common strategy to incorporate such interactions is by tuning the chemical functionality of the end group of the polymer chain, thereby generating telechelic polymers. In this scheme, either one or both end groups of each substituent polymer can be functionalized, generating a variety of different architectures for supramolecular assembly in telechelic polymer blends.

When ionic groups are installed as the polymer end groups, they can provide tunable, localized interactions that significantly influence the self-assembly behavior and can stabilize ordered morphologies even at relatively low molecular weights.^17,20^ In recent years, numerous computational approaches,^21–24^ such as the Random Phase Approximation (RPA)^25^ and Self-Consistent Field Theory (SCFT),^26–28^ have been adopted to investigate the phase behavior of telechelic polymer blends. These computational insights reveal that the self-assembly behavior of telechelic systems differs fundamentally from that of traditional BCPs, exhibiting a rich variety of nanostructures and phase transitions governed by the balance between ionic bonding strength and segregation strength, given by κ = *h*_ab_/(*χN*). Here, *h*_*ab*_ is the Gibbs free energy for associating the oppositely charged polymer chain ends, which is directly related to the ionic interaction strength. Elliott and Fredrickson developed a theoretical framework to describe supramolecular assembly in telechelic polymer blends.^29^ Their result showed lamellar structures at low values of χ*N*, which cannot be observed in conventional BCPs. Their phase diagram showed two extremes: (1) when κ ≪ 1, the ionic interaction is insufficient to prevent macrophase separation between the polymer chains, and self-assembly is not observed; and (2) when κ ≫ 1, the ionic interaction is sufficient to maintain the association of end groups, and the phase behavior is similar to what is observed for BCPs. In between these extremes, when κ ∼ 1, the ionic interaction strength is similar in magnitude to χ*N*, and the phase behavior of the system is highly dependent on temperature as both *h*_ab_ and χ are temperature-dependent properties. This increased control over the polymer self-assembly imparts stimuli-responsive properties to telechelic blends, with several previous reports of temperature-dependent structural transitions that are significantly more tunable than what can be achieved in conventional BCP systems.^18,30^

However, only a few experimental works have validated the computational predictions due to challenges in both material design and synthesis. Russell et al.^31^ and Zhang et al.^32^ studied blends of two difunctionalized homopolymers, generating “ABAB” blend architectures, and demonstrated that telechelic blends were capable of supramolecular self-assembly into nanostructures with long-range order and small periodicities. The nanostructures were highly dependent on temperature, where dissociation of the end groups at high temperatures, due to decreases in *h*_AB_, led to macrophase separation. Guillén Obando et al.^33^ reported a simple route to prepare supramolecular block copolymers using difunctionalized-sulfonated polystyrene (PS) and monofunctionalized-aminated polydimethylsiloxane (PDMS), generating an “ABA” blend architecture. While the small-angle X-ray scattering (SAXS) profiles exhibited a broad primary peak, the system did not microphase separate into nanostructures with long-range order potentially due to the high dispersity of the PDMS polymer (*Đ* ∼ 1.7) or impurities within the PS. Huh et al.^30^ investigated how blending ratio and temperature affect the supramolecular assembly behavior of monofunctionalized-aminated polyisoprene and difunctionalized-sulfonated PS, or “ABA” blends. They show that phase transitions can be induced by both changes in temperature as well as blending ratio. Xie et al. recently reported the effect of end group functionality on the nanostructure of monofunctionalized-sulfonated PS and monofunctionalized-PDMS, known as “AB” blends.^34^ By tuning the basicity of the PDMS end group, effectively changing *h*_AB_, they were able to observe a variety of distinct nanostructures in their telechelic blends.

However, several synthetic challenges exist when introducing ionic end groups to polymer chains. Since the end group fraction is usually low and high molar-purity is required to achieve sufficient end group association, rigorous purification of the polymers is necessary.^35^ Additionally, due to the presence of side reactions or kinetic issues, achieving both low Đ and high functionality is often contradictory for a given polymer synthesis.^36–39^ As the ionic end groups are usually strong acids or bases, they are not compatible with all polymer backbones, such as Si–O–Si, and can lead to decomposition of the polymer chain during end group installation. Following polymer synthesis, the nanostructure is largely influenced by the chosen processing conditions, which must be carefully controlled to obtain sufficient end group association.^40^ Finally, once the self-assembled system is formed, the associated end group, which now serves as the junction between homopolymer blocks in the supramolecular self-assembly, must maintain stability under acidic/basic and high-temperature conditions.

Here, we study the effect of blend properties such as polymer molecular weight and mixing ratio on the phase behavior of telechelic blends. We propose a new synthetic method to prepare monofunctionalized-piperidine-terminated PDMS (PipPDMS). The functionalized initiator was synthesized through a simple two-step reaction using commercially available reagents with high yields. PDMS was subsequently prepared via living anionic polymerization, affording polymers with high end group functionality and low *Đ* after deprotection. A series of monofunctionalized-sulfonated PS, purified to ensure 100% end group functionality, were blended with PipPDMS to prepare a set of telechelic “AB” blends, Figure 1. The oppositely charged end groups enable heteroassociation of −SO_3_H/-Pip functionality present on PS and PDMS chains as well as self-association between homopolymers, *i*.e., formation of −SO_3_H/-SO_3_H and -Pip/-Pip aggregates. The ratio of heteroassociation to self-association strongly influences the resulting blend nanostructure. The phase behavior of the blends was characterized by SAXS at various temperatures, revealing the formation of long-range ordered structures and several temperature-induced phase transitions. For blends that formed disordered phases, RPA was used to extract the degree of heteroassociation, *z*, and effective interaction parameters, χ_eff_. We found that all blends that exhibit disordered phases have a quantitatively similar value of *z*. The effect of mixing ratio was also probed on blend phase behavior. Insights from SAXS were used to construct a phase diagram to compare with theoretical predictions. By precisely tuning the mixing ratio, we were able to induce phase transitions and achieve a variety of ordered nanostructures with long-range order.

**Figure 1:**
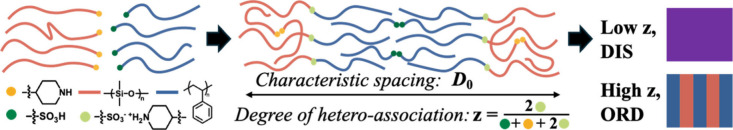
Schematic of telechelic AB blend system used in this study.

## Materials and Methods

### Materials

Styrene, *sec*-butyllithium (*sec*-BuLi), 1,3-propane sultone, 1,1-diphenylethylene (DPE), acetic acid, Karstedt’s catalyst (Pt ∼ 2% in xylene), benzoic acid, hexamethylcyclotrisiloxane (D_3_), potassium iodine (KI), trifluoroacetic acid (TFA), NaHCO_3_, d-chloroform, silver trifluoroacetate (AgTFA), trans-2-[3-(4-*tert*-butylphenyl)-2-methyl-2-propenylidene]­malononitrile (DCTB), triethylamine (TEA), chlorotrimethylsilane (TMSCl), lithium bis­(trimethylsilyl)­amide (LiHMDS, 1.0 M in THF), hydrochloric acid (37%), chloroform, *N*,*N*-dimethylformamide (DMF), dichloromethane (DCM), acetonitrile (ACN), toluene, tetrahydrofuran (THF), petroleum ether (PE), ethyl acetate (EA), and methanol were purchased from Sigma-Aldrich. *tert*-Butyl-4-methylenepiperidine-1-carboxylate and dimethylmethoxysilane were purchased from Fisher Scientific. Toluene, THF, D_3_, and styrene were distilled under high vacuum before use; all other chemicals were used as received.

### Preparation of Functionalized Initiator

Dimethylmethoxysilane (10.57 g, 2 equiv, 101.4 mmol) and 200 μL of Karstedt’s Catalyst was added to a solution of *tert*-butyl 4-methylidenepiperidine-1-carboxylate (10.0 g, 1 equiv, 50.7 mmol) in toluene (200 mL). The solution was stirred under Ar at 50 °C overnight. The solution was concentrated, purified by silica gel chromatography PE/EA to obtain *tert*-butyl 4-((ethoxydimethylsilyl)­methyl)­piperidine-1-carboxylate (14.0 g, 46.4 mmol, yield 91.6%), **2** in Scheme 1, as a colorless oil. Acetic acid (200 uL) was added to a solution of **2** (8.0 g, 1 equiv, 26.5 mmol) in THF (50 mL)/H_2_O (10 mL). The solution was stirred at room temperature overnight. The solution was concentrated, purified by silica gel chromatography by PE/EA, 4/1 to obtain *tert*-butyl 4-((hydroxydimethylsilyl)­methyl)­piperidine-1-carboxylate (7.1 g, 26.0 mmol, yield 97.9%), **3** in Scheme 1, as a white solid.

**Scheme 1:**
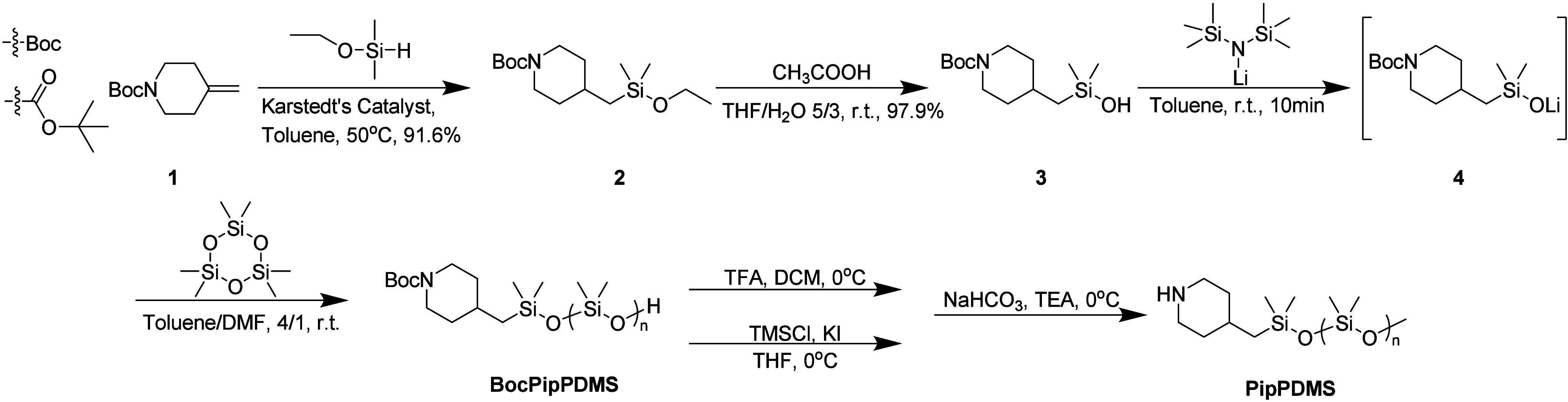
Synthesis of Functionalized Initiator and Preparation of Piperidine Functionalized PDMS, PipPDMS

### Polymerization of Piperidine Functionalized PDMS

LiHMDS (4.3 mL, 4.3 mmol, 1.02 equiv) was added to a solution of **3** (1.2 g, 4.23 mmol, 1.0 equiv) in toluene (6 mL). The solution was stirred at room temperature for 10 min under Ar. Then, the solution was added to a solution of D_3_ (30 g, 134.5 mol, 33 equiv) in toluene (80 mL). 20 mL DMF was added to the solution as a promoter. The solution was stirred at room temperature for 1 h under Ar. Then, an excess amount of benzoic acid was added to quench the reaction. The solution was concentrated, extracted by methanol three times to yield 27 g of BocPipPDMS as a colorless oil (*M*_n_ = 6700 g/mol, Đ = 1.085). The product was deprotected to get 10 g PipPDMS as a colorless oil, see SI.

### Preparation of PS/PDMS Blends

PS and PDMS were dissolved in toluene, a nonselective solvent, as a 10w t% solution. The PDMS solution was added to the PS solution; the amount was based on the end group molar ratio. The solution was stirred at room temperature for 30 min then poured into a Teflon dish. The solution was evaporated slowly at ambient temperature and pressure for at least 16 h to get wet samples. The samples were further dried in the vacuum oven at 35–40 °C for 24 h to remove the residual toluene. Following drying, the samples were transparent thin films or gummy samples, depending on the volume fraction of PDMS.

### Characterizations and Measurements

Nuclear Magnetic Resonance (NMR) Spectroscopy: ^1^H NMR spectroscopy and ^19^F NMR were obtained from Bruker Avance 500 MHz and Bruker Avance 400 MHz spectrometers. Chloroform-*d* (≥99.8 atom %D, anhydrous) was selected as NMR solvent.

Fourier transform infrared (FTIR) spectra were obtained with a NICOLET iS50 FTIR spectrophotometer using the transmission mode.

Size exclusion chromatography (SEC): Gel permeation chromatography (GPC) was performed using the following setup: an Agilent Technologies 1260 Infinity II pump with two inline Agilent PLgel (part #: PL1113-6300) columns, Wyatt Technology mini-DAWN light scattering, and Agilent 1260 Infinity II RI detectors, using HPLC grade toluene as the mobile phase, at a flow rate of 1 mL min^–1^. Values of PDMS d*n*/d*c* (−0.0806) were determined using Wyatt’s ASTRA software assuming 100% mass recovery from the GPC columns.

MALDI-TOF measurements: MALDI-TOF was measured via Bruker ultraflex III using DCTB as matrix and AgTFA as ionizing agent. The sample preparation and measurement followed the method reported in ref 35.

Small-angle X-ray scattering (SAXS) measurements: The PS/PDMS blends were prepared by sealing by Kapton polyimide tape. Temperature-dependent SAXS measurements were performed using a Xeuss 3.0 system. Measurements were performed every 10 °C from 50 to 150 °C with an exposure time of 600 s in standard mode. Samples were allowed to equilibrate at each temperature for 20 min before measurement. The resulting 2D scattering patterns were isotropic and were azimuthally integrated into 1D profiles using XSACT Pro advanced data analysis software. Background intensities from the Kapton tape were subtracted from the total intensity. The SAXS intensity, *I*(*q*), was recorded as a function of the magnitude of the scattering wavevector, defined as $q=\frac{4\pi }{\lambda }\;\sin\left(\frac{\theta }{2}\right)$ where θ is the scattering angle and λ is the wavelength of the X-ray.

## Results and Discussion

### Polymer Synthesis and Blend Preparation

The formation of ordered self-assembled morphologies in telechelic polymer blends depends critically on maintaining high end-group fidelity and ensuring that the end groups remain stable under high-temperature, acidic, and basic conditions. Therefore, we developed a new route to synthesize PDMS from a functionalized initiator to ensure 100% end group functionality before deprotection. Scheme 1 shows the synthetic route of the functionalized initiator, which has not been previously reported. Commercially available piperidine, **1**, was reacted with an excess amount of dimethyl­(ethoxy)­silane to give **2**. Deprotection of **2** gave the desired silanol, **3**, as our functional initiator. To generate our end-functionalized PDMS, we deprotonated initiator, **3**,with LiHMDS to reveal the active oxyanion needed for effective chain propagation. Unlike the related ring opening polymerization (ROP) of D_3_, reported by Fuchise via 1,3-trimethylene-2-n-propylguanidine (TMnPG), our polymerization is not sensitive to water.^41–43^ Additionally, this functional initiator approach eliminates challenges with isolation by eliminating large amount of biproduct formation, such as OH-PDMS-OH, which is difficult to separate from BocPipPDMS via conventional chromotography.^41^ After the addition of LiHMDS, residual water in the functionalized initiator is quenched, and the silanol group is activated, while the piperidine group remains protected by the tert-butoxycarbonyl (Boc) group. Following generation of the active initiator, **4**, the monomer, D_3_, and promoter, DMF, were added for chain propagation. In this synthesis, the chain-end functionality is 100% as chain coupling cannot occur in living anionic polymerization and each chain will have one Boc-Piperidine group from the functionalized initiator. Careful control of conditions such as reaction time and conversion suppresses excessive backbiting. Following polymerization, the Boc-Piperidine terminated PDMS was deprotected, by two methods. The acid method follows the classic deBoc condition and uses TFA/DCM to deprotect the end group. A neutral method reported by Merck was also applied,^44^ and both methods yield similar results. The reaction was performed at 0 °C to suppress the hydrolysis of the backbone. Due to the sensitivity of the Si–O–Si linkages, ∼10% of oligomer from the Si–OH side is generated during the deprotection which is observed in GPC and ^1^H NMR, see Figures S12–S14. The NMR peak corresponding to the formation of the oligomers is consistent with Fuchise’s report. Further optimization of deprotection conditions is needed to reach 100% fidelity in future. However, the relative ^1^H NMR peaks of chain end and PDMS backbone does not change after deprotection, Figure S8.

Table 1 shows the properties of the PS and PDMS polymers used in this study. Three PS polymers with different molecular weights and 100% end group functionality were synthesized via anionic polymerization and purified by silica gel chromatography; details are provided in the SI.^38,45^ In order to study blend phase behavior, precise measurement of the mixing ratio, *r*, is needed. Polymer molecular weight is measured via ^1^H NMR for PDMS and MADLI-TOF for PS. Normalized degree of polymerization (*N*_*i*_) is calculated according to

1 $$N_{i}=\frac{M_{i}}{\rho_{i}N_{A}\upsilon_{ref}}$$

where *N*_A_ is Avogadro’s number, ν_ref_ is the reference volume taken as 0.1 nm^3^, and *M*_*i*_ and ρ_*i*_ are the molecular weight and density of styrene and D3 monomers, respectively. Due to the strong interaction between the GPC column and the acidic end group of PS, *Đ* of PS is calculated using MALDI data since the intensity is proportional to the number of chains with specific mass according to^46^

2 $$Đ=\frac{\underset{i}{\sum }x_{i}M_{i}^{2}}{{\left(\underset{i}{\sum }x_{i}M_{i}\right)}^{2}}$$

where *M*_*i*_ is the molecular weight of the polymer chain and *x*_*i*_ is the fraction of *M*_*i*_ which is calculated from the fraction of total intensity. SAXS data for each pure polymer shows a featureless profile at ambient temperature for PDMS and at various temperatures for PS, Figures S15–S16.

**Table 1:** Characteristics of Functionalized PS and PDMS

polymer	molecular weight(kg/mol)	**N**_**i**_ ^a^	**Đ**
PS5.5K	5.5^b^	69	1.01^b^
PS8.9K	8.9^b^	111	1.01^b^
PS12.5K	12.5^b^	156	1.01^b^
PDMS2.6K	2.6^b^	35	1.28^c^
PDMS5.8K	5.8^d^	78	1.09^c^
PDMS10.5K	10.5^d^	142	1.08^c^

a Normalized degree of polymerization is calculated based on ν_ref_ = 0.1 nm^3^.

b *M*_w_, Calculated based on MALDI.

c Determined by GPC.

d *M*_n_, Determined by ^1^H NMR.^43^

Table 2 shows the properties of polymer blends used in this study. PS/PDMS mixing ratio, *r*, is calculated according to the molar ratio of chain ends. Total normalized chain length of the blend, *N*, is calculated according to

$$\{\begin{array}{c} N=r\times N_{PS}+N_{PDMS}\;for\;r>1\\ N=N_{PS}+N_{PDMS}\;/r\;for\;r<1 \end{array}$$ 3

where *N*_*i*_ is taken from Table 1. The fraction of PS in the blends (*f*_PS_) is calculated by *N*_PS_/*N*. Characteristic spacing, *D*_0_, is taken from SAXS measurements according to, $D_{0}=\frac{2\pi }{q_{0}}$ , where *q*_0_ is the position of the primary peak and is reported as the stable temperature, *T*_s_, which will be discussed in the subsequent sections. For structure, C refers to hexagonally packed cylinders, L+C refers to coexisting phases of lamellae and hexagonally packed cylinders, Dis refers to disordered structure, and L refers to lamellae. The structure is also reported at *T*_s_ for each blend.

**Table 2:** Properties of Polymer Blends

Polymer 1	Polymer 2	*r* ^a^	*N* ^b^	*f*_*PS*_ ^c^	*D*_0_ ^d^ (nm)	*T*_s_ (°C)	structure^d^
PS5.5K	PDMS2.6K	1	133	0.66	16.2	150	Dis
PS8.9K	PDMS2.6K	1	187	0.76	17.2	130	Dis
PS12.5K	PDMS2.6K	1	245	0.82	16.6	100	Dis
PS5.5K	PDMS5.8K	1	188	0.47	27.4	120	Dis
PS8.9K	PDMS5.8K	1	242	0.59	24.8	150	Dis
PS12.5K	PDMS5.8K	1	300	0.67	23.3	150	Dis
PS8.9K	PDMS5.8K	0.83	262	0.54	27.8	150	Dis
PS8.9K	PDMS5.8K	1.1	256	0.61	30.1	150	Dis
PS8.9K	PDMS5.8K	1.3	285	0.65	35.2	150	Dis
PS8.9K	PDMS5.8K	1.5	313	0.68	34.6	130	Dis
PS8.9K	PDMS10.5K	1	323	0.44	32.7	150	L+C
PS8.9K	PDMS10.5K	1.3	366	0.50	32.2	130	L+C
PS8.9K	PDMS10.5K	1.5	394	0.54	31.1	90	L
PS8.9K	PDMS10.5K	2	465	0.61	37.38	90	Dis

a Molar ratio calculated by mole fraction of end groups, see Eq. 3.

b Calculated based on *r* and ν_ref_.

c Calculated based on *r* and ν_ref_.

d Taken at *T*_s_.

### Effect of Molecular Weight on Nanoscale Structure

Figure 2 shows the SAXS profiles of equimolar blends, *r* = 1, of PDMS2.6K, Figure 2a, and PDMS5.8K, Figure 2b, with PS5.5K, PS8.9K, PS12.5K at 100 °C. The formation of −SO_3_H/-Pip ion pairs association was confirmed via FTIR and ^1^H NMR spectroscopy, see Figures S17 and S18. Based on the SAXS profiles, all six telechelic blends form disordered morphologies due to the presence of a broad scattering peak indicative of concentration fluctuations. The location of the primary scattering peak, *q*_0_, generally decreases as *N* increases, corresponding to an increase in the characteristic length-scale of the concentration fluctuations as the polymer components get larger. For example, in the blend prepared with PS5.5K, *D*_0_ is 16.2 nm for blends prepared with PDMS2.6K at 100 °C, Figure 2a, while *D*_0_ is 27.4 nm for the blends prepared with PDMS5.8K, Figure 2b, at the same temperature. However, when the PDMS molecular weight is fixed, *D*_0_ is not very sensitive to changes in the molecular weight of the PS block. For example, *D*_0_ is between 16.2 and 17.2 nm for the three prepared blends in the PDMS2.6K series and *D*_0_ ranges from 23.3 to 27.8 nm in the PDMS5.8K series. Also, the full width at half-maximum (fwhm) of the scattering peaks, which characterizes the degree of long-range order in the system, are not correlated with *N*, see Figure S27.^47^ For disordered BCPs, as the system approaches an order disorder transition (ODT) by increasing *χN*, the peak becomes sharper and the fwhm decreases.^48^ Therefore, increasing *N* in a BCP results in a sharpening of the scattering peaks. In the PDMS5.8K series, the fwhm of the peaks are independent of *N*, indicating that our blends are not approaching the ODT as the PS molecular weight increases. Previously experimental result of ABA telechelic blend systems showed that the volume fraction of homopolymers undergoing heteroassociation also exerts a significant influence on *D*_0_.^30^ Therefore, we believe that tuning the PS molecular weight does not significantly alter the volume fraction of heteroassociation, which leads to similar nanoscale structures (i.e., similar characteristic spacings and degree of long-range order) for each blend series. Previous experimental work from Xie and co-workers demonstrated that for AB telechelic blend systems, *D*_0_ is consistently larger than that of a pure BCP with the same molecular weight and volume fraction due to the presence of unassociated and self-associated homopolymer chains, which swell the microdomains.^34^ Therefore, our telechelic blends behave similarly to blends of AB BCPs doped with homopolymers A and B, where the supramolecule, i.e., BCP, is segregated to the interface between the two bulk homopolymers leading to swelling of the domains.^49^ In our system, we believe that insufficient heteroassociation between the PS and PDMS end groups essentially limits the concentration of supramolecules in our blends. Therefore, the concentration of supramolecules is not high enough to overcome the barrier for microphase separation, and instead only disordered phases are observed.

**Figure 2:**
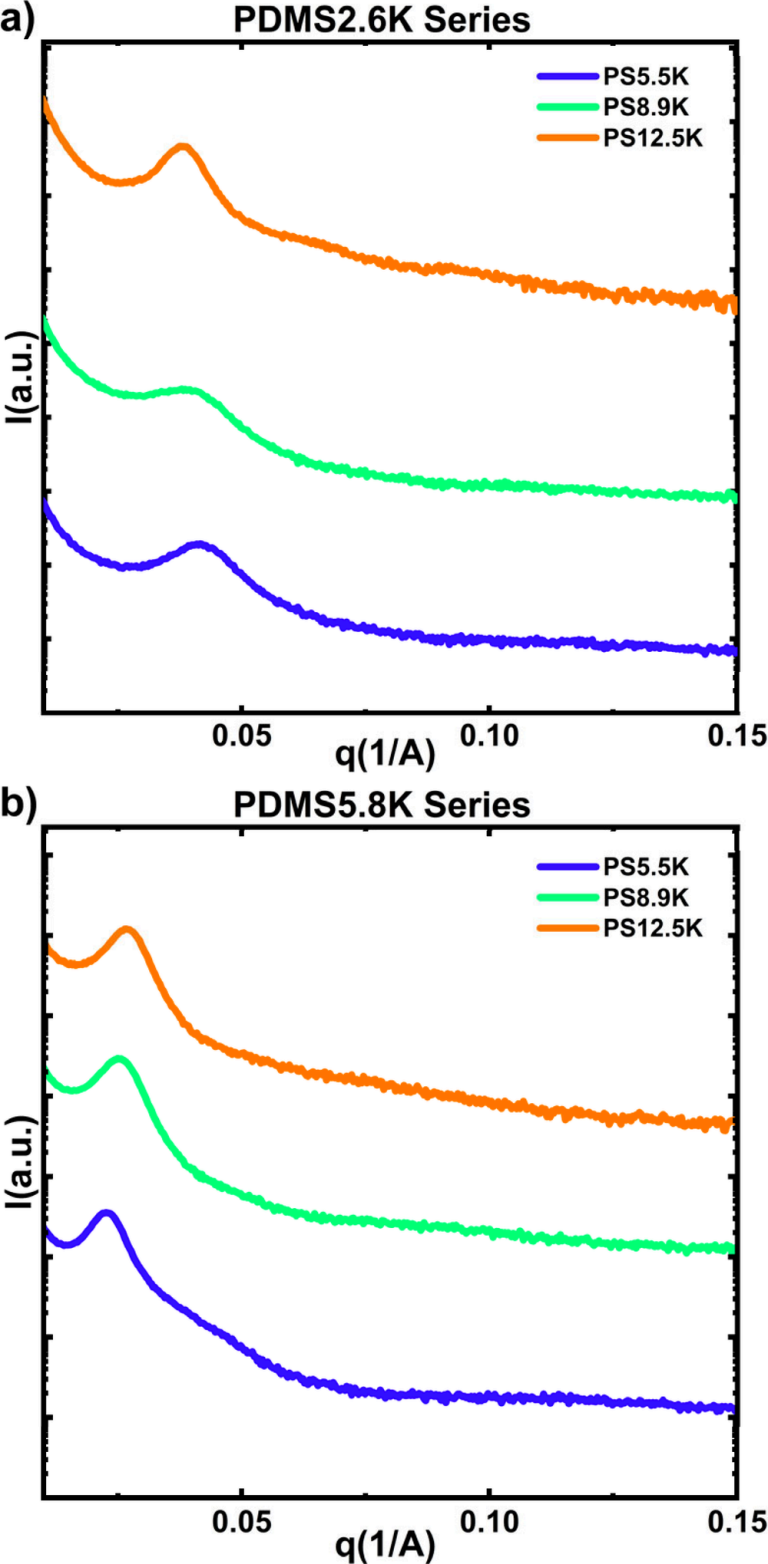
SAXS profiles of (a) PDMS2.6K and (b) PDMS5.8K equimolar blend with varying molecular weights of PS at 100 °C.

Figure 3 shows SAXS results of equimolar, *r* = 1, PDMS2.6K polymer blends at different temperatures for PS5.5K, Figure 3a, PS8.9K, Figure 3b, and PS12.5K, Figure 3c. Overall as temperature increases, the scattering intensity also increases although two types of temperature-induced transitions are observed. There is a change in total intensity as the temperature increases, with a shift in *q*_0_ without a significant change in peak shape, as observed in Figure 3a for the PDMS2.6KPS5.5K blends. From 50 to 90 °C, *q*_0_ moves to higher values of *q* indicating a decrease in characteristic length-scale, then upon further heating from 90 to 150 °C, *q*_0_ moves to lower values of *q* indicating an increase in characteristic length-scale. These transitions are reversible with temperature, see Figure S20. Additionally, the fwhm of the peak is constant with temperature; see Figure S27. We define a stable temperature (*T*_s_) as the highest temperature at which *q*_0_ and fwhm do not change and report these values in Table 2. Therefore, for the PDMS2.6KPS5.5K blend, *T*_s_ is 150 °C, or the highest accessible temperature. However, in the blends with higher molecular weight PS, such as PDMS2.6KPS8.9K, as temperature increases the peak shape changes with an increase in fwhm at 130 °C indicating that the system becomes more disordered as temperature increases. This trend is more pronounced in the PDMS2.6KPS12.5K blend where we see the scattering peak broaden and move to lower *q* at 100 °C corresponding to a significant increase in the fwhm. As temperature continues to increase, the peak shifts to lower values of *q* and eventually disappears for the blend. All changes in scattering are reversible for *T* ≤ *T*_s_, while the transitions observed for *T* > *T*_s_ are irreversible; see SI. The irreversible change in scattering behavior occurs because the supramolecule dissociates and enters the bulk phase, causing swelling in the domains. As the supramolecule continues to dissociate with increasing temperature due to the temperature dependence of *h*_AB_, there is not sufficient heteroassociation, and finally, macrophase separation occurs. These observations show the effect of κ on *T*_s_. As κ decreases due to an increase in *N*, the balance between ionic interactions and segregation strength is insufficient to maintain association and leads to macrophase separation. The temperature dependent SAXS profiles for the PDMS5.8k series are provided in Figure S19. Overall, the PDMS5.8K series are significantly more stable with temperature.

**Figure 3:**
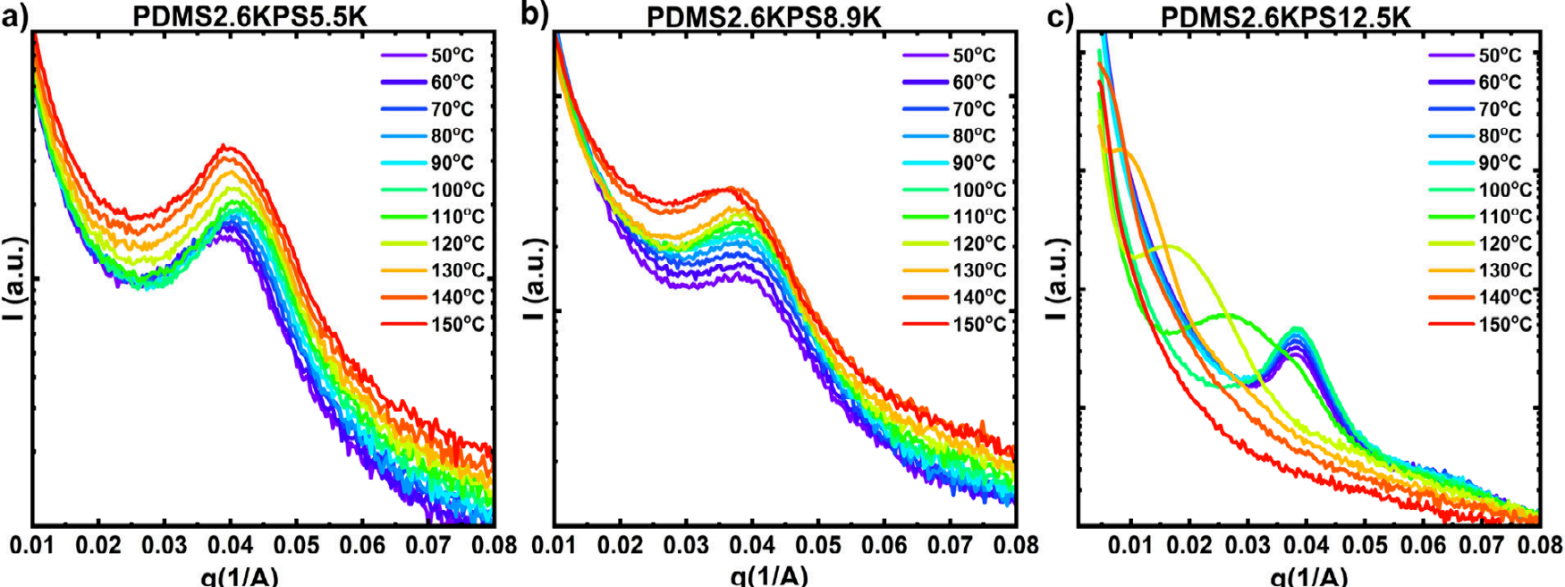
SAXS profiles of PDMS2.6K equimolar blend at different temperatures with PS (a) 5.5 K, (b) 8.9 K, and (c) 12.5 K.

To further understand the thermodynamics and nanoscale structure of the blends, a model reported by Tanaka on supramolecular assembly is used to extract *z* and χ_eff_ from the disordered SAXS profiles according to^25^

4 I(q)=C·T(q)

Here, *C* corresponds to the X-ray contrast of the system that arises from differences in electron and material density. *T*(*q*) is calculated according to the RPA

5 $$T\left(q\right)=\frac{1}{S\left(q\right)/W\left(q\right)-2\chi_{\mathrm{eff}}}$$

where

6 $$S\left(q\right)=S_{AA}^{o}\left(q\right)+S_{BB}^{o}\left(q\right)+2S_{AB}^{o}\left(q\right)$$

7 $$W\left(q\right)=S_{AA}^{o}\left(q\right)S_{BB}^{o}\left(q\right)+{\left[S_{AB}^{o}\left(q\right)\right]}^{2}$$

The structure factor has been derived in Tanaka’s model, which takes the following form

8 $$S_{AA}^{o}\left(q\right)=NaD\left(aQ\right)f_{PS}$$

9 $$S_{BB}^{o}\left(q\right)=NbD\left(bQ\right)\left(1-f_{PS}\right)$$

10 $$S_{AB}^{o}\left(q\right)=\frac{N}{2}\left[D\left(Q\right)-a^{2}D\left(aQ\right)-b^{2}D\left(bQ\right)\right]z$$

where

11 $$Q={\left(qR_{g}\right)}^{2}=\frac{Nq^{2}l^{2}}{6}$$

12 $$D\left(x\right)=\frac{2\left(\exp\left(-x\right)-1+x\right)}{x^{2}}$$

Here, *N* is the degree of polymerization of the associated BCP given by *N* = *N*_PS_ + *N*_PDMS_, *l* is the statistical segment length, taken as 5 Å, *a* is the fraction of the A block (PS) on a single BCP chain, *a* = *N*_PS_/*N*, *b* is the fraction of B block (PDMS) on a single BCP chain, *b* = *N*_PDMS_/*N*, *f*_PS_ is the total volume fraction of A block in the polymer blend, and *D*(*x*) is the form factor of Gaussian coil. A background function is used to correct for imperfect subtraction of background and takes the form

13 $$I_{background}=y_{0}+A_{background}q^{y}$$

The parameters are fitted using lower and higher angle data at 50 °C to obtain three parameters, *y*_0_, *A*_background_, and *y*. The averaged intensity between 0.06 A^–1^ and 0.15 A^–1^ at different temperatures is used to scale up the background intensity with changing temperature. Equations 13 and 4 are applied to the temperature dependent SAXS profiles for all blends and the fits are provided in Figures S29–S34.

Figure 4 shows the results from the RPA model and plots *z*, Figure 4a, and χ_eff_, Figure 4b, as a function of temperature for the PDMS2.6K series (solid line) and the PDMS5.5K series (dashed line). The data is reported only for *T* ≤ *T*_s_ for each blend. Surprisingly, the fitted value of *z* is quantitatively similar for all blends and is independent of temperature at *z* ∼ 0.4, translating to only 40% of the oppositely charged end groups present within the blend associating. The values of *z* for the PDMS5.8K series are slightly lower than those for the PDMS2.6K series, indicating that association scales with number of available end groups. This is consistent with the theoretical result from Fredrickson; if the electrostatic strength of the association cannot overcome the entropic penalty of mixing due to high *χN*, the volume fraction of association will be low, i.e., *z* = 0.5.^27^ This low value of association prevents stabilization of an ordered nanostructure and results in the formation of only disordered phases. Additionally, the constant value of *z* across all blends studied indicates that these blends form similar nanostructures as observed by the similar scattering features. Notably, the PDMS5.8KPS5.5K blend has the lowest value of heteroassociation, which explains the decrease in temperature stability where *T*_s_ = 120 °C. Figure 4b plots χ_eff_ for each blend as a function of temperature. While χ_eff_ is rather constant with temperature for each blend, we see that it is highly dependent on *N*. The values of χ_eff_ for the PDMS2.6K series are significantly higher than those of the PDMS5.8K series matching previous studies on using RPA to extract values of χ_eff_ from BCPs have shown that χ_eff_ decreases with increasing *N*.^50^ However, for the PDMS2.6K series we observe that χ_eff_ increases with increasing *N* (due to changes in PS molecular weight), while for the PDMS5.8K series, χ_eff_ decreases with increasing *N*. Finally, the fitted values of χ_eff_ are lower than previous reports for PS-*block*-PDMS BCPs.^51^ We hypothesize that these discrepancies arise due to the variation in temperature stability of the blends, where χ_eff_ is higher for the blends with *T*_s_ < 150 °C. We believe that the quantitative differences in the value of χ_eff_ arise due to the simplification of the chosen RPA model, which neglects the effects of dispersity and self-association of the charged end groups. Previous theory calculated that lamellar phases would only be stabilized if *z* ≥ 0.5, which quantitatively agrees with our results.^27^ Therefore, despite the inconsistencies with χ_eff_, the consistent value of *z* across all blends studied indicates that the disordered phase arises in supramolecular polymer blends when there is insufficient heteroassociation. Increasing *N* and studying blends with PDMS5.8K and PS8.9K also results in the formation of disordered phases likely due to insufficient values of *z*, see Figure S39.

**Figure 4:**
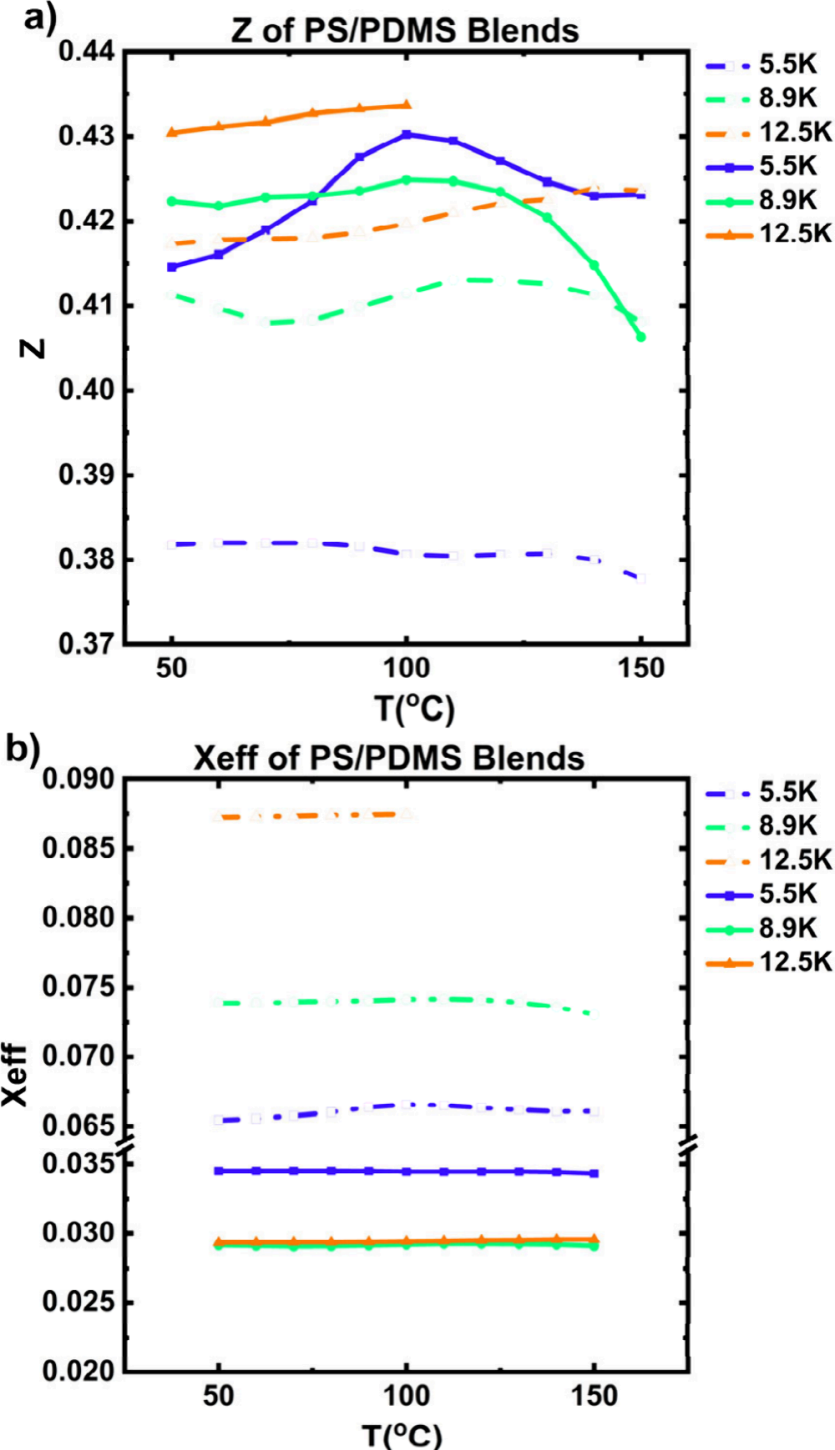
Results from RPA fits for a) *z* and b) χ_eff_ as a function of temperature where the solid line refers to the PDMS2.6K series and the dashed line refers to the PDMS5.8K series.

### Effect of Mixing Ratio on Phase Behavior

To further increase segregation strength while ensuring sufficient PS end groups for association, new blends of PDMS10.5KPS8.9K were prepared at different values of *r*. Figure 5 **s**hows SAXS profiles for blends of PDMS10.5KPS8.9K at 70 °C, Figure 5a, and 120 °C, Figure 5b. Higher order scattering peaks are observed for blends with 1 ≤ *r* ≤ 1.5, and the primary scattering peaks occur at similar values of *q*_0_, indicating that the characteristic length-scale of the well-defined nanostructure is solely dependent on the molecular weight of the constituent homopolymers rather than their relative ratios. The temperature dependent SAXS profiles for the blends are provided in Figures S22–S25. For blends with *r* ≤ 1.5, the scattering peaks become sharper as the temperature is increased, while for the blend with *r* = 2, the scattering peaks become broader as temperature increases. At the lowest concentrations of PDMS10.5K, the nanoscale structure is sensitive to temperature, and we observe macrophase separation where the scattering peaks fully disappear at 140 °C for *r* = 1.5 and 120 °C for *r* = 2. These results indicate that when the balance between acid and base pairs is asymmetric, the degree of heteroassociation is strongly affected by temperature. As temperature increases, the electrostatic interactions that stabilize the ordered phase weaken and lead to macrophase separation. This effect is exaggerated when there is an asymmetric amount of acid and base pairs, which limits the potential for rearrangement of the associating bonds.

**Figure 5:**
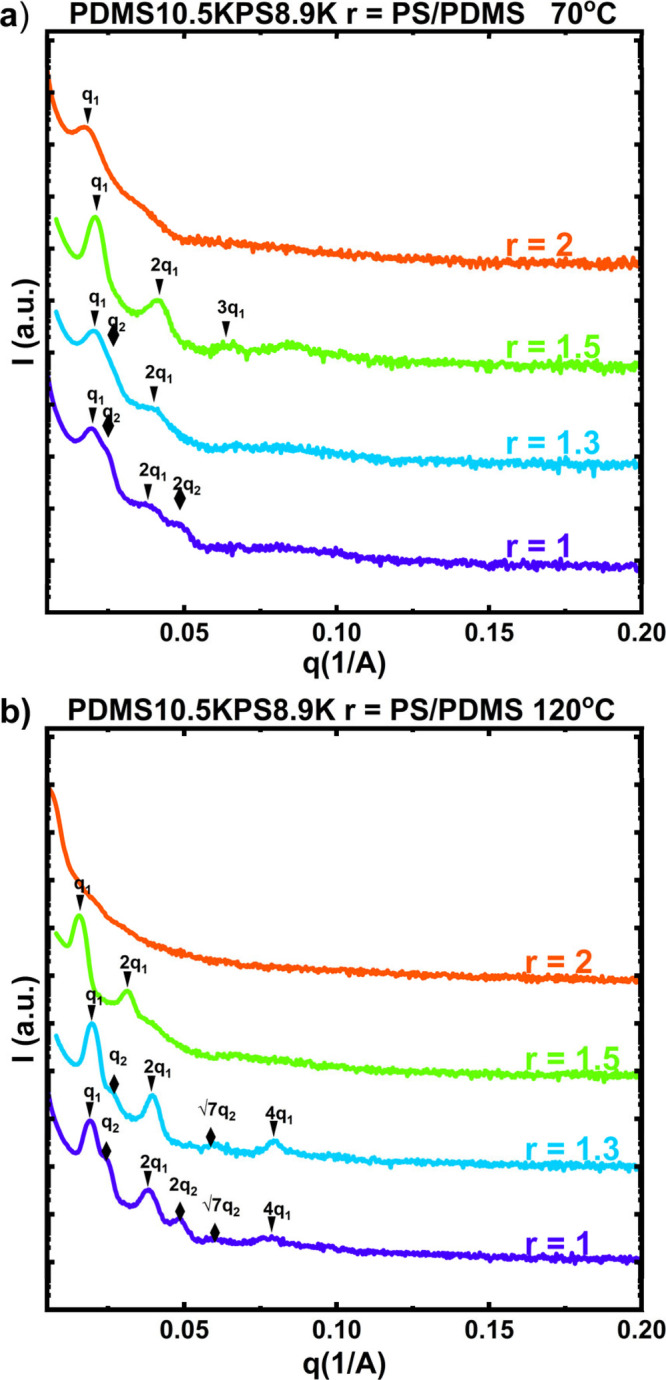
SAXS profiles of PDMS10.5KPS8.9K blends with different mixing ratios, *r*, at a) 70 °C and b) 120 °C.

The mixing ratio also has a strong effect on blend nanoscale structure. At mixing ratio of *r* = 2, the system forms a disordered phase, due to the insufficient association between end groups. As the concentration of PS is decreased to *r* = 1.5, the blend forms a lamellar phase. At mixing ratios of *r* = 1 and 1.3, the blends form coexisting lamellar and cylindrical phases based on the ratio of primary to higher order scattering peaks, see Figure S26 and Table S1. Based on the strength and location of the primary scattering peaks, the lamellar phase is the majority phase with a larger characteristic length-scale with *D*_0,*L*_ = 32.9 nm versus *D*_0,*C*_ = 27.0 nm, when *r* = 1. Previous experimental studies of telechelic polymer blends also observed coexistence between ordered phases.^30,52^ This hybrid structure is also stable with respect to temperature. The Porod invariant, Q*, of the scattering data can be used to quantify the relative volumes of a specific phase given by^53^

14 $$Q^{*}=\int_{0}^{\infty }q^{2}I\left(q\right)dq\propto \phi_{1}\phi_{2}{\left(\rho_{1}-\rho_{2}\right)}^{2}$$

However, due to the limited understanding of the system’s microstructure, the low-*q* and high-*q* regions of the scattering cannot be reliably extrapolated, and therefore the full scattering invariant (Q*) from *q* = 0 to *∞* cannot be obtained. Instead, we estimate the relative volume fractions of the lamellar and cylindrical phases by analyzing the appropriate *I*(*q*)*q* ^2^ vs *q* ranges for each structure. We apply Gaussian peak fitting to the relevant scattering peaks and calculate the invariant for each peak (*Q*) using eq 14 where the bounds of the integral are taken from the scattering data. The ratio of these peak-specific invariants provides a quantitative measure of the relative amounts of each phase, as shown in Figures S35–S38. The integration of the fitting range (*Q*) is calculated using the fitted Gaussian peaks and the results are shown in Figure 6. Figure 6a shows *Q* as a function of temperature for the lamellar and cylindrical phases for the *r* = 1 blend. As the temperature increases, both *Q*_L_, shown in blue, and *Q*_*C*_, shown in yellow, increase, but the primary phase remains lamellar, as indicated by *Q*_L_ > *Q*_C_ over the entire temperature window. Figure 6b shows *Q* as a function of temperature for the lamellar and cylindrical phases for the *r* = 1.3. Like the equimolar blends, *Q*_*L*_ increases with increasing temperature and is significantly larger than *Q*_*C*_ at all temperatures. However, *Q*_*C*_ remains constant with respect to temperature. These changes in structure are reversible in this temperature window as *T*_*s*_ = 150 °C. Figure 6c plots the ratio of the invariants, $\phi_{L}=\frac{Q_{L}}{Q_{c}}$ , as a function of temperature to quantify the relative volume fractions of each phase. In both blends, ϕ_L_ > 1, indicating that the lamellar phase is the majority phase. However, ϕ_L_ is higher for the *r* = 1.3 blend, shown in yellow, compared to the *r* = 1 blend, shown in blue, at all temperatures. For the *r* = 1 blend, ϕ_L_ increases steadily with increasing temperature from ∼3 at 50 °C to ∼5 at 150 °C indicating moderate growth of the lamellar phase with increasing temperature. However, ϕ_L_ is highly dependent on temperature for the *r* = 1.3 blend. When the temperature is under 80 °C, ϕ_L_ for the *r* = 1.3 blend is quantitatively similar to that of the *r* = 1 blend. Once the temperature is increased above 80 °C, ϕ_L_ increases dramatically and reaches a peak of 19 at 120 °C, followed by a decrease above 120 °C. Therefore, the lamellar phase at *r* = 1.3 is sensitive to high temperatures, similar to what is observed for the *r* = 1.5 sample.

**Figure 6:**
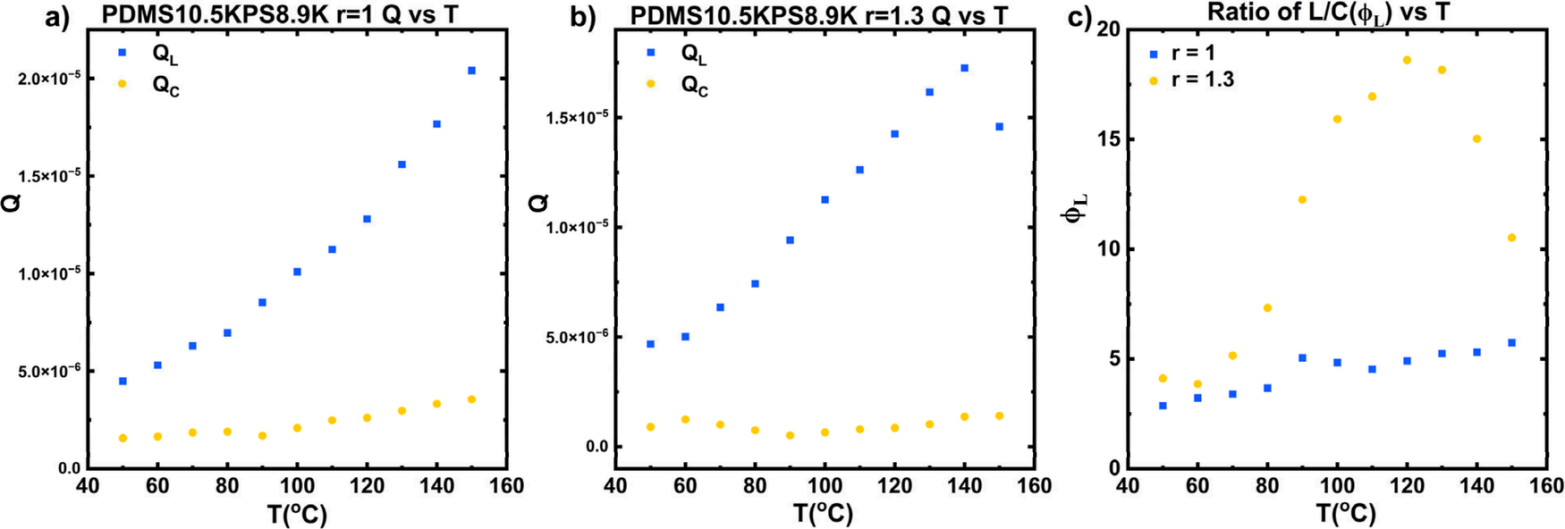
Integration of *I*(*q*)*q* ^2^ vs *T*: values of Q for the lamellar (*Q*_L_, blue) and cylindrical (*Q*_*C*_, yellow) for the a) *r* = 1 and b) *r* = 1.3 blends. c) ϕ_L_ = *Q*_L_/*Q*_*C*_ vs *T* for *r* = 1 (blue) and *r* = 1.3 (yellow) blends.

Insights from the scattering profiles were used to construct a phase diagram for the PDMS10.5KPS8.9K blends at different temperatures and mixing ratios and is presented in Figure 7. Here, we use *f*_PS_ to quantify blend composition in order to enable comparison with previously developed phase diagrams for BCPs and telechelic blends. At the lowest composition of *f*_PS_ studied, corresponding to the *r* = 1 blend, we observe coexisting lamellar and cylindrical phases. At *r* = 1, the lamellar phase is the majority phase and as *f*_PS_ increases, corresponding to the *r* = 1.3 blends, the relative volume fraction of the lamellar phase also increases. Upon increasing *f*_PS_ to 0.54, representing the *r* = 1.5 blend, we observe lamellar phases at low temperatures followed by an irreversible transition to a macrophase separated system at 120 °C. At the highest value of *f*_PS_ studied, corresponding to the *r* = 2 blends, we only observe disordered phases at low temperatures followed by a transition to a lamellar phase at *T* = 100 °C and finally to macrophase separation at *T* = 110 °C. These results are consistent with both Huh and Noro’s report that pure lamellae is not observed at stoichiometric ratios of telechelic polymers with -SO_3_H/-NH_2_ ion pairs due to inefficient heteroassociation. Instead, they see asymmetric phase diagrams similar to our observations in Figure 7. Noro’s TEM result showed that unassociated polymers will enter the ordered phases, while heteroassociated supramolecules will distribute along the interfaces to stabilize the nanostructure. However, they saw ordered phases when the mole fraction of -SO_3_H was higher than that of -NH_2_ and macrophase separation when the mole fraction of -NH_2_ is higher than that of -SO_3_H. We see the opposite trends and attribute these differences to the increased p*K*_a_ value between -Pip and -NH_2_, which alters the equilibrium of the acid/base association.^34^

**Figure 7:**
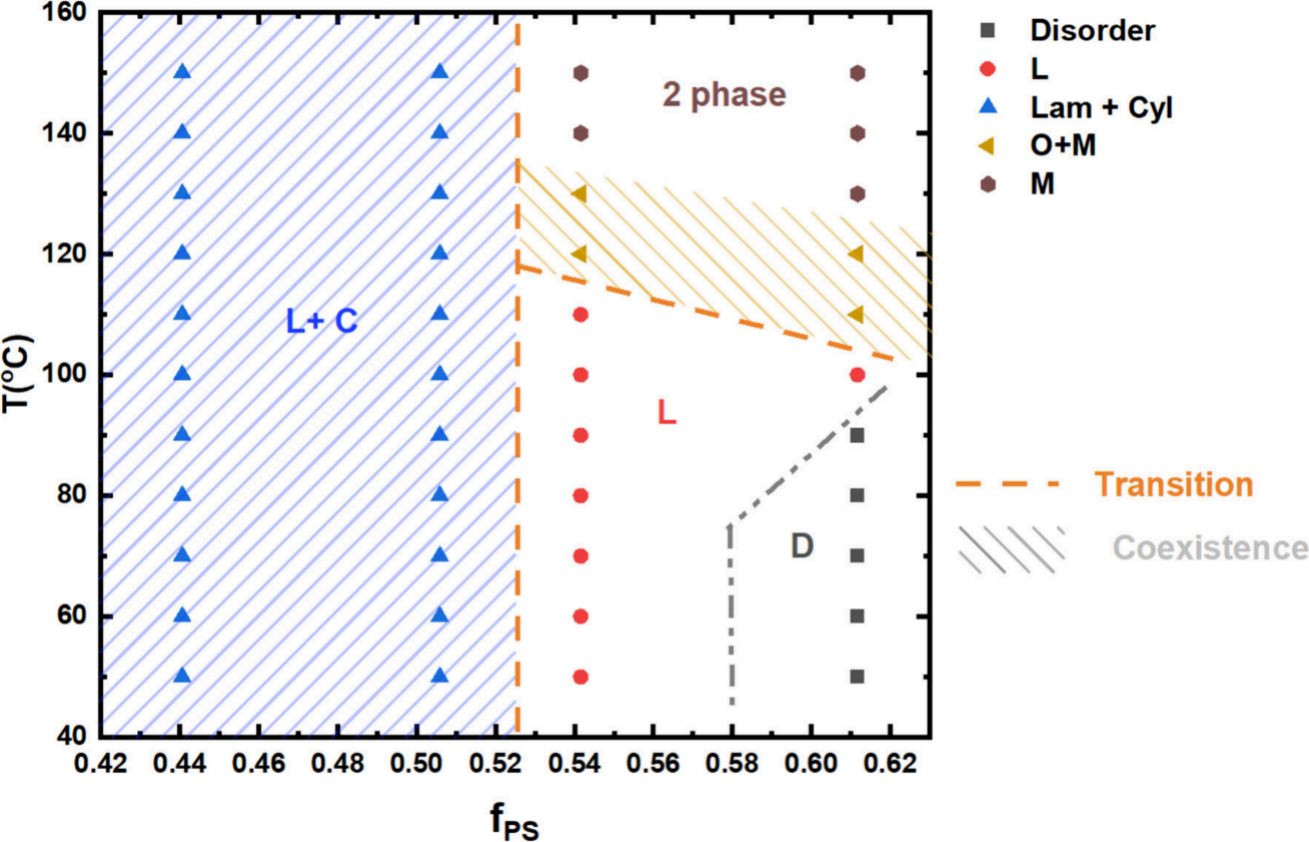
Phase diagram of PDMS10.5KPS8.9K blends as a function of PS volume fraction (*f*_PS_) where D refers to disordered phase, L refers to lamellar phase, L+C refers to coexisting lamellar and cylindrical phases, M refers to macrophase separation and O+M refers to coexisting ordered phases with macrophase separation.

Based on the presented SAXS results and previous experimental results from AB and ABA systems,^30^ a hypothesis for the mechanism is proposed. Besides *χN* and *r*, the relative ratio of heteroassociation and self-association plays a vital role in determining the blend nanostructure. Due to the low concentration of functionalized end groups in the equimolar state and corresponding kinetic barriers,^34^ not all telechelic polymers will undergo heteroassociation. Thus, many highly polar end groups do not reach the AB interface and instead remain surrounded by themselves in a nonpolar environment, and previous theory shows that these end groups will instead undergo self-association.^29^ The low dielectric constants of the polymer backbone preferentially encourage self-association of the highly polar end groups to minimize the Gibbs free energy. Additionally, compared to the solution state, a large kinetic barrier exists for the charged end groups within the blend to diffuse through the domains to the AB interface. Therefore, the resulting structure is largely influenced by the number of initial heteroassociated pairs formed during solvent casting, which is directly controlled by the chosen processing method. For example, previous work has shown that employing freeze-drying methods can significantly increase the number of initial heteroassociated pairs formed.^40^ As temperature increases, dissociation of the heteroassociated pairs begins, and the enthalpic repulsion between the polymers, characterized by *χN*, tends to reintroduce free chains into the PDMS or PS domains. While *N* is low, *χN* is weak and concentration of end groups is high. Due to the high concentration of nearby oppositely charged end groups, as temperature decreases the heteroassociation is recovered, and therefore this transition is reversible as evident by a constant value of *z* presented in Figure 4a for *T* < *T*_s_. For our systems where *T*_s_ = 150 *°C*, we are unable to reach the temperature required to dissociate the ion pairs due to experimental limitations. We hypothesize that at sufficiently high temperatures, we would observe a sharp decrease in *z* and induce macrophase separation in all blends due to the high value of *χN* between PS and PDMS. As the number of end groups and the compositional asymmetry increases (for blends with *r* ≠ 1), excess chain ends form self-associated pairs to minimize the system’s free energy in all observed nanostructures. For a lamellar system, as temperature increases, dissociation of heteroassociated end groups occurs, and these end groups are reintroduced into the PS and PDMS domains into self-associated pairs. As the degree of heteroassociation decreases, macrophase separation occurs, indicated by *T*_s_ < 150 *°C*, as there are insufficient supramolecules to stabilize the ordered phase. This transition is irreversible, as upon cooling, the end groups that were located at the AB interface are now participating in self-association and cannot undergo heteroassociation, similar to what is observed in the PDMS2.6KPS12.5K system shown in Figure S19. Therefore, the resulting blend nanostructure is highly dependent on the balance between heteroassociation and self-association of end groups, which is largely influenced by both *r* and temperature.

## Conclusions

In conclusion, we show how molecular weight and mixing ratio can be used to effectively tune the nanostructure of telechelic polymer blends. By developing a novel functionalized initiator, we are able to synthesize a library of PipPDMS polymers with low dispersity and >90% end group functionality after deprotection. AB telechelic blends were prepared with monofunctionalized PipPDMS and monofunctionalized PS polymers and their phase behavior was studied via temperature dependent SAXS. For blends that form disordered phases, we use a model based on RPA to extract the degree of heteroassociation and show that blends with only 40% of associated end groups are unable to overcome the ODT and form ordered nanostructures. However, high molecular weight blends exhibited a variety of hybrid ordered nanostructures, and by precisely tuning the mixing ratio, we were able to induce phase transitions and achieve a variety of ordered nanostructures with long-range order. A phase diagram developed from our results agrees qualitatively with previous theoretical predictions and experimental results. We propose a mechanism for the observed phase behavior that balances the ionic interactions of the polymer end groups with *χN*. Future work will investigate the effect on end group concentration by studying ABA telechelic systems to enable direct comparison with the AB telechelic systems studied here.

## Supplementary Material



## Notes

Materials and Methods, additional SAXS data and fitting results (PDF)
