# Supplementary material for: Supramolecular Assembly of End-Functionalized Polystyrene and Polydimethylsiloxane with Hybrid Nanostructures
Source: Macromolecules. 2026 Mar 17;59(7):4485–96. doi: 10.1021/acs.macromol.5c03429 (PMC13085851; doi:10.1021/acs.macromol.5c03429)
Supplement: Supplementary file 1 [file ma5c03429_si_001.pdf]

Supporting Information for

Supramolecular Assembly of End-Functionalized Polystyrene and Polydimethylsiloxane with Hybrid Nanostructures

Jingchao Qin[1], Whitney S. Loo[1]*

[1]Department of Chemical and Biological Engineering, University of Wisconsin-Madison, Madison, WI, 53706

*Corresponding Author: wloo@wisc.edu

Table of Contents

Sample preparation and characterization ... 3

Synthesis of $-SO_3H$ functionalized PS ... 3

Matrix-assisted laser desorption/ionization time of flight (MALDI-TOF) mass spectroscopy of PS ... 4

Deprotection of BocPiperidinePDMS ... 6

Proton nuclear magnetic resonance (^1H NMR) spectroscopy ... 7

Size exclusion chromatography ... 12

Structural Characterization ... 24

Small Angle X-Ray Scattering (SAXS) of Homopolymers ... 24

FTIR and NMR characterization of blends ... 25

Small Angle X-Ray Scattering (SAXS) result of PDMS/PS Blends ... 26

SAXS Fitting Result ... 34

Full Width at Half Maximum of PDMSPS Blends ... 34

Porod invairant of PDMSPS blends ... 35

Total Derivation of Porod Invariant vs T ... 35

Random Phase Approximation (RPA) Fitting Result ... 36

Gaussian Peak Fitting Result: Porod Invariant ... 39

PDMS5.8kPS8.9k Blends: Effect of Mixing Ratio ... 41

Reversibility of Phase Transitions ... 43

Sample preparation and characterization

Synthesis of -SO_3H functionalized PS

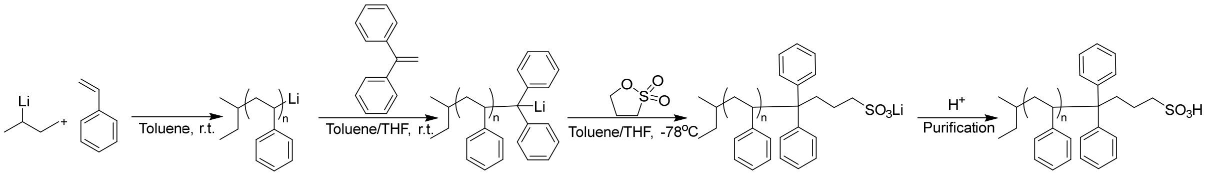

To a solution of 25mL styrene in degassed THF or Toluene was added 15mL n-Butyl Lithium inside the glovebox. The solution is stirred overnight to ensure all of the moisture is quenched. THF/Toluene was then distilled via high vacuum technique into air-free reactor. Purified styrene (22mL, 192mmol, 55eq) was added to 220mL Toluene inside glovebox. 1.4M sec-Butyl lithium (2.5mL, 3.5 mmol, 1eq) was then added as initiator. The solution is stirred overnight at ambient temperature inside the glovebox. Then DPE (618uL, 3.5 mmol, 1eq) and 150mL THF were added to the solution and stirred for 3h. The solution was terminated by 1,3-Propanesultone (0.64g, 5.25mmol, 1.5eq) at -78°C. Then solution was concentrated, purified by silica gel chromatography DCM, DCM/MeOH 8/1 to get 18g PS-SO_3Na as white solid. The PS-SO_3Na was dissolved into DCM and precipitated into MeOH with droplet HCl/MeOH solution three times to get 14g of PS as white solid.

Matrix-assisted laser desorption/ionization time of flight (MALDI-TOF) mass spectroscopy of PS

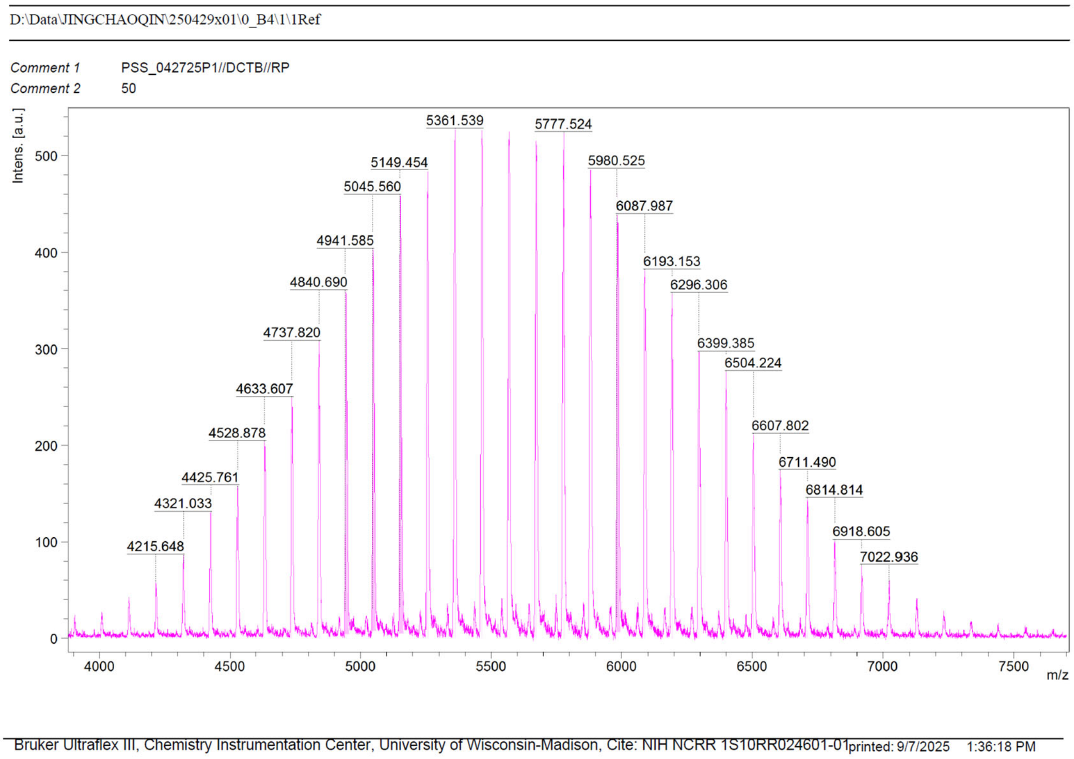

Figure S1. MALDI-TOF result of PS5.5K

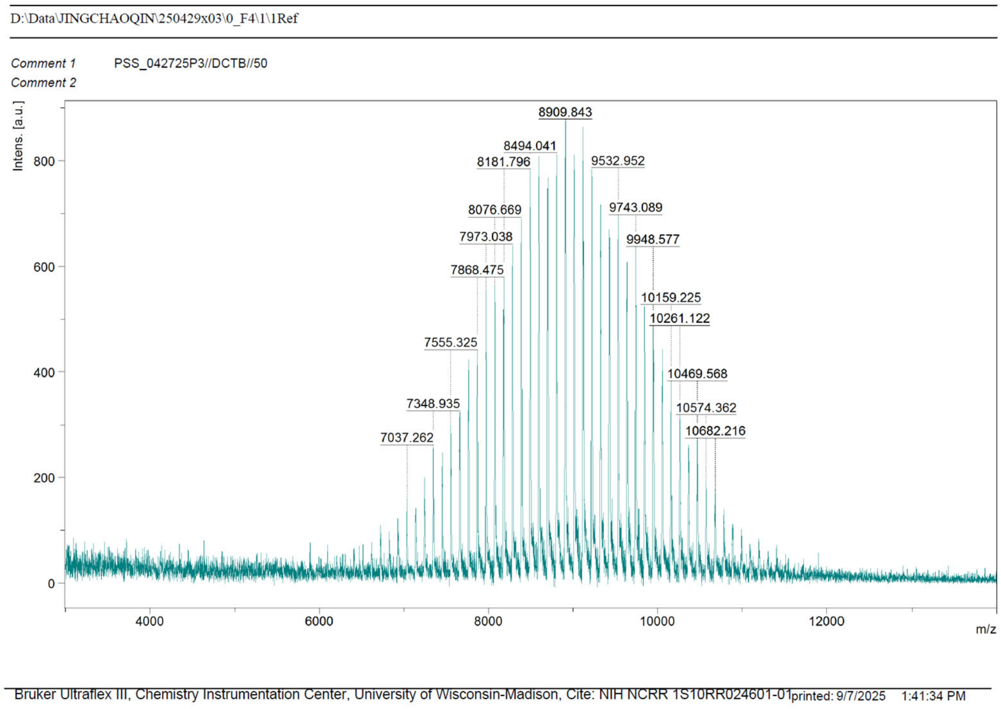

Figure S2. MALDI-TOF result of PS8.9K

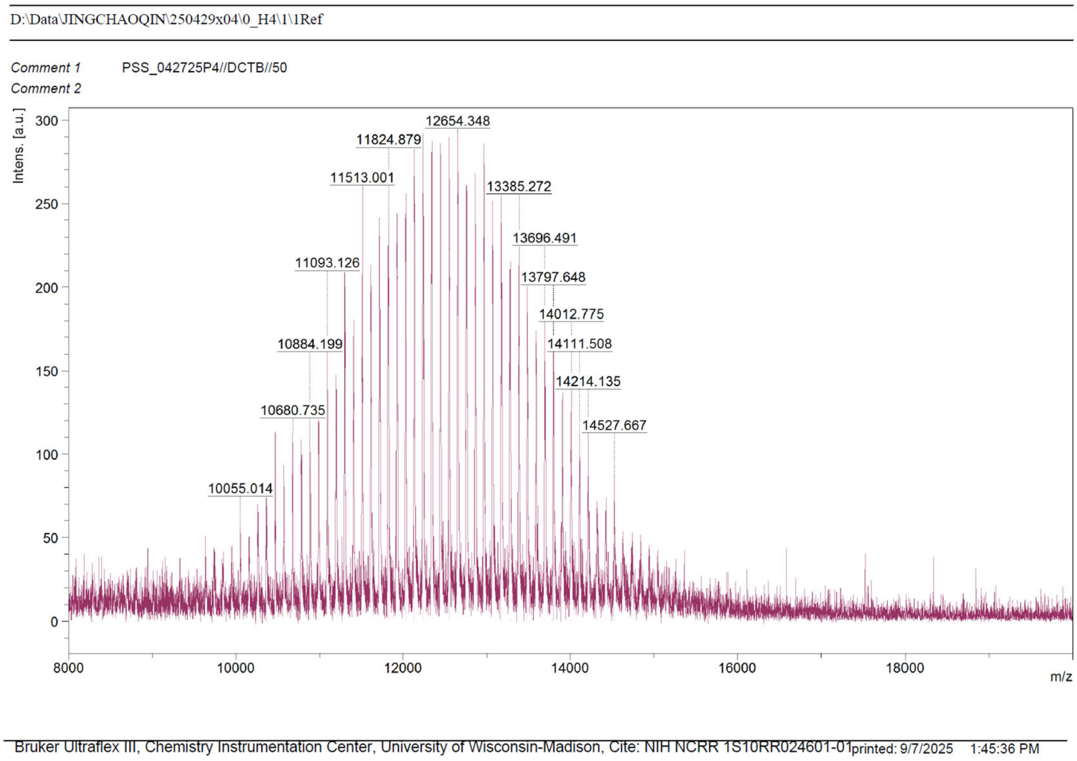

Figure S3. MALDI-TOF result of PS12.5K

Deprotection of BocPiperidinePDMS

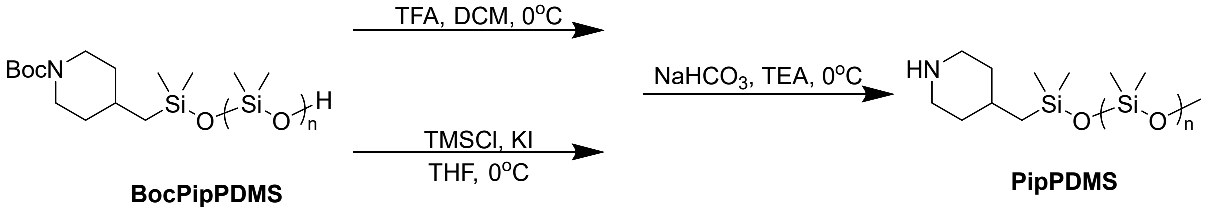

Acid Method

To a solution of BocPiperidinePDMS2.6K (20g) in 40mL DCM was added 20mL TFA. The solution was stirred at 0°C for 20min. The solution is added to saturated $NaHCO_3$ aqueous solution, followed by adding 3mL of TEA. The organic layer is then dried by Na_2SO_4, conentrated to get colorless oil. The oil was wash by MeOH/ACN, 5/1 three times, concentrated to get PipPDMS (11g, 55% yield) as colorless oil.

Neutral Method

To a solution of BocPipPDMS10.5k(20g) in THF(100mL) was added TMSCl(2g, 18.4mmol) and KI(3.05g, 18.4mmol). The solution is stirred at 0°C for 30min. The solution was then poured into the aqueous Na_2SO_3 solution, extracted by DCM. The organic layer was then washed by $NaHCO_3$ aqueous solution, followed by adding 2mL TEA and conentrated to get colorless oil. The oil is washed in same method as acid method to get PipPDMS(15.2g, 76% yield) as colorless oil.

Proton nuclear magnetic resonance (^{1}H NMR) spectroscopy

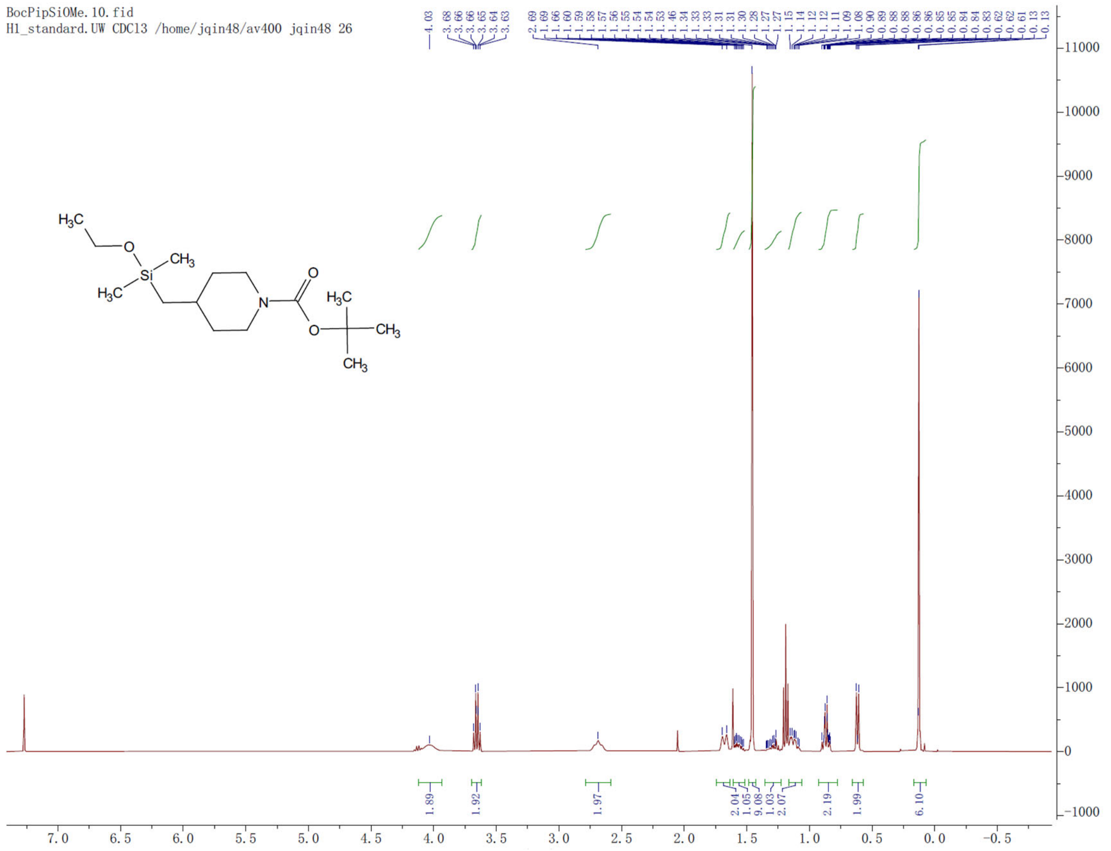

Figure S4. NMR of tert-butyl 4-((ethoxydimethylsilyl)methyl)piperidine-1-carboxylate

1H NMR (400 MHz, CDCl3) δ 4.03 (s, 2H), 3.70 – 3.61 (m, 2H), 2.93 – 2.55 (m, 2H), 1.68 (d, J = 13.3 Hz, 2H), 1.61 – 1.51 (m, 1H), 1.46 (s, 9H), 1.36 – 1.23 (m, 1H), m 1.15 – 1.07 (m, 2H), 0.91 – 0.81 (m, 2H), 0.62 (d, J = 6.9 Hz, 2H), 0.13 (s, 6H).

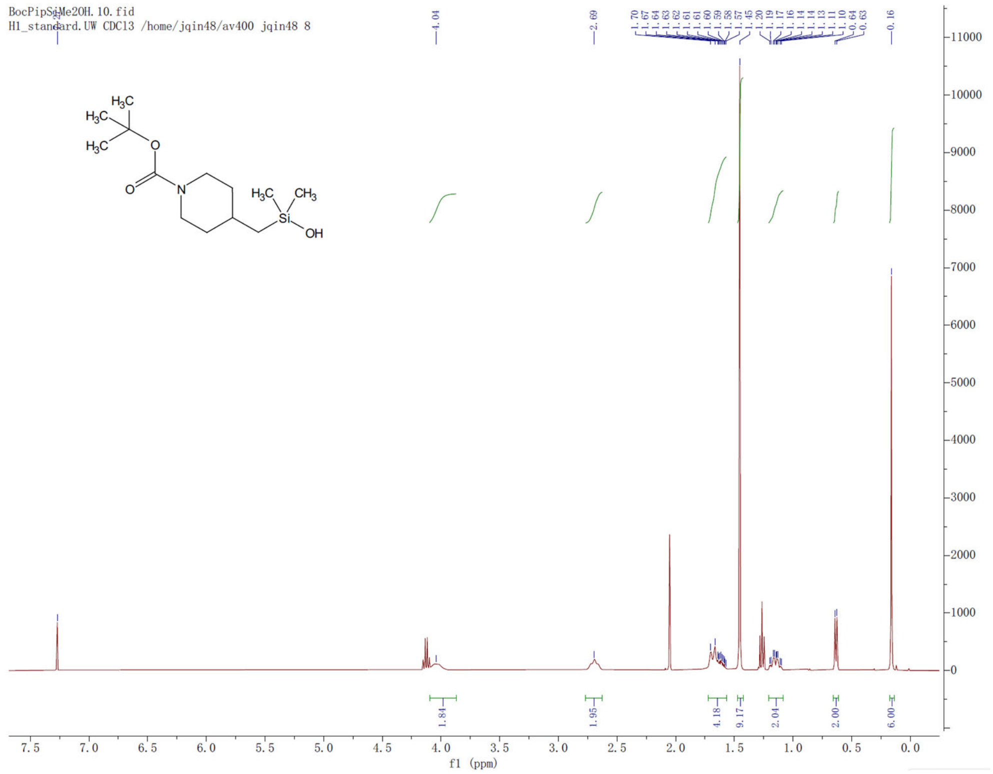

Figure S5. NMR of tert-butyl 4-((hydroxydimethylsilyl)methyl)piperidine-1-carboxylate

1H NMR (400 MHz, CDCl3) δ 4.04 (s, 2H), 2.79 – 2.60 (m, 2H), 1.76 – 1.54 (m, 4H), 1.45 (s, 9H), 1.22 – 1.10 (m, 2H), 0.63 (d, J = 6.8 Hz, 2H), 0.16 (s, 6H).

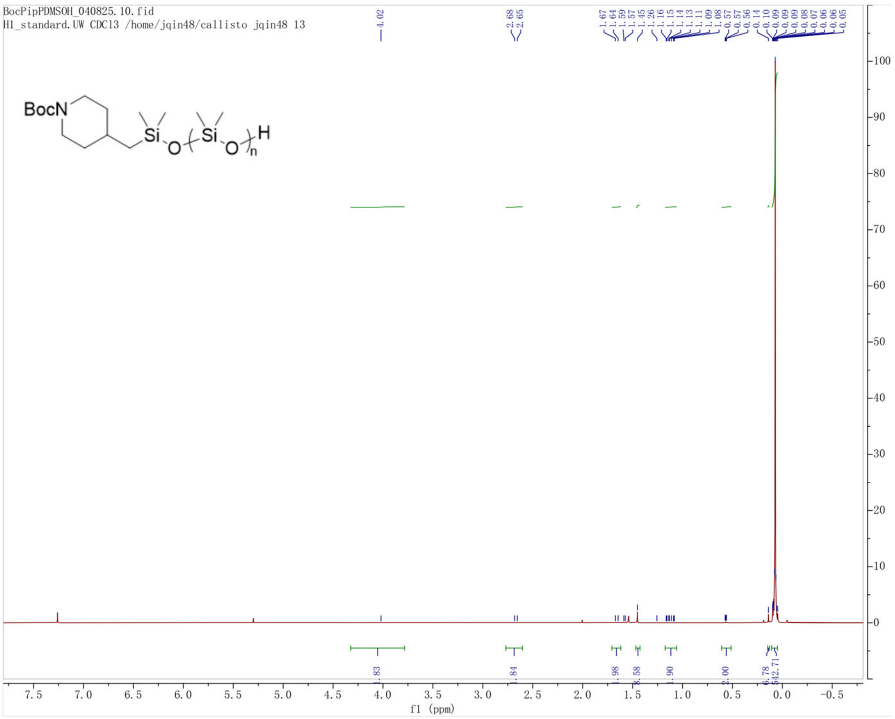

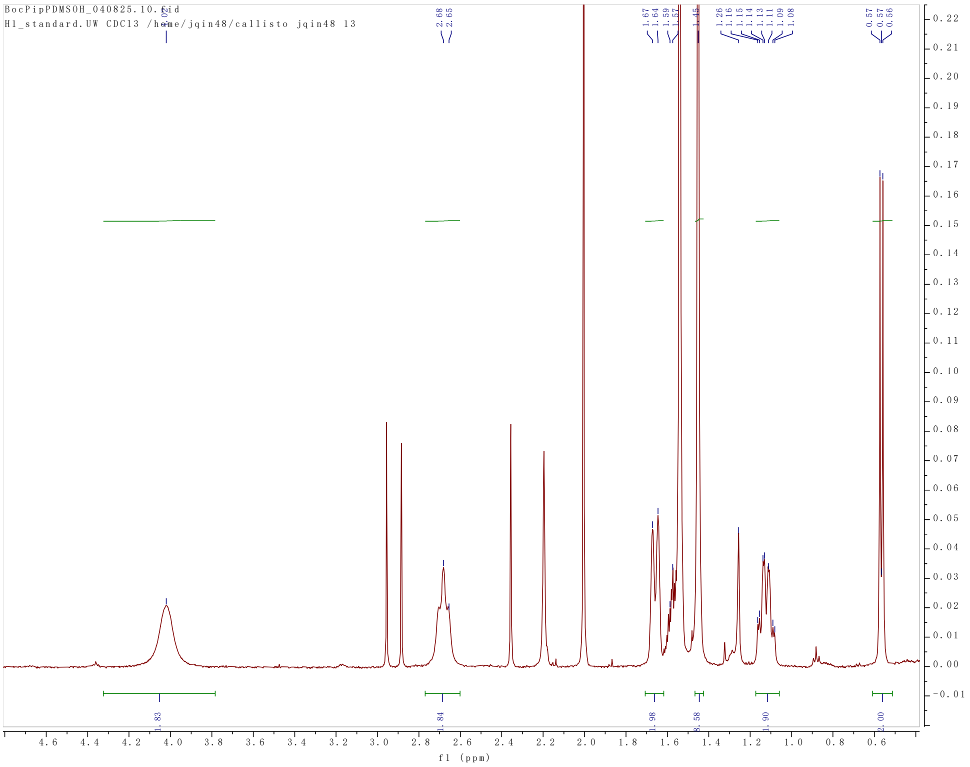

Figure S6. NMR of BocPiperidinePDMS and magnified end group peaks

^{1}H NMR (500 MHz, $CDCl_3$) δ 4.02 (s, 2H), 2.79 – 2.57 (m, 2H), 1.66 (d, *J* = 13.2 Hz, 2H), 1.62 – 1.56 (m, 1H), 1.45 (s, 9H), 1.27 – 0.97 (m, 2H), 0.57 (d, *J* = 6.9 Hz, 2H), 0.14 (s, 6H), 0.12 – -0.04 (m, 577H).

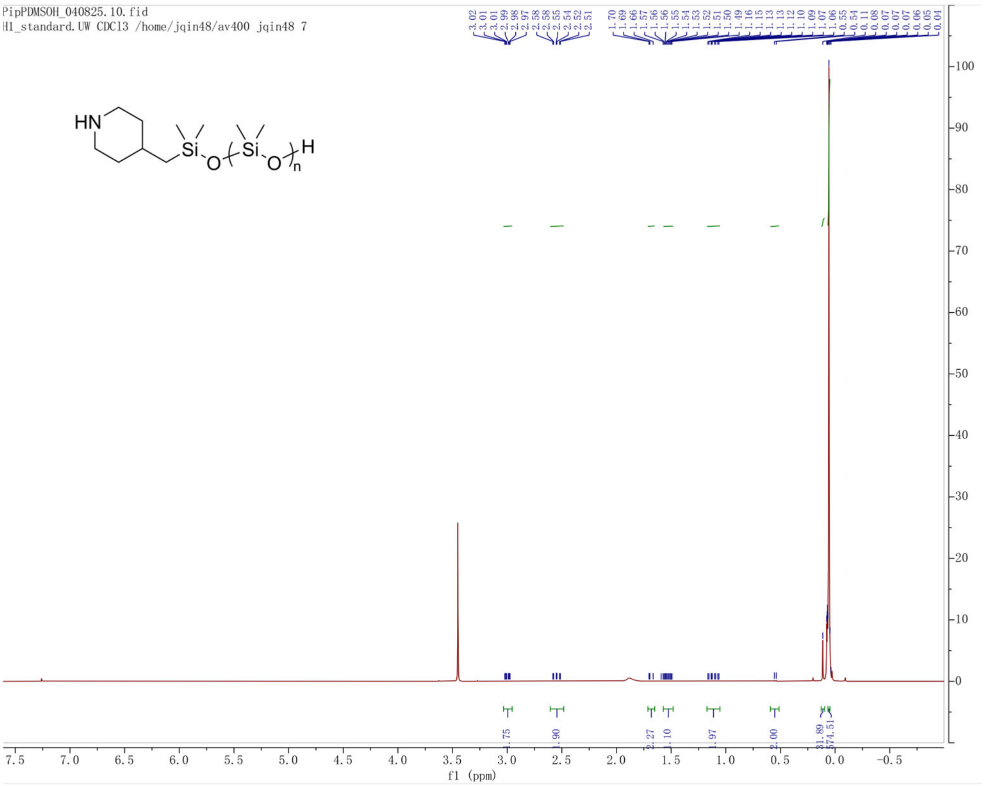

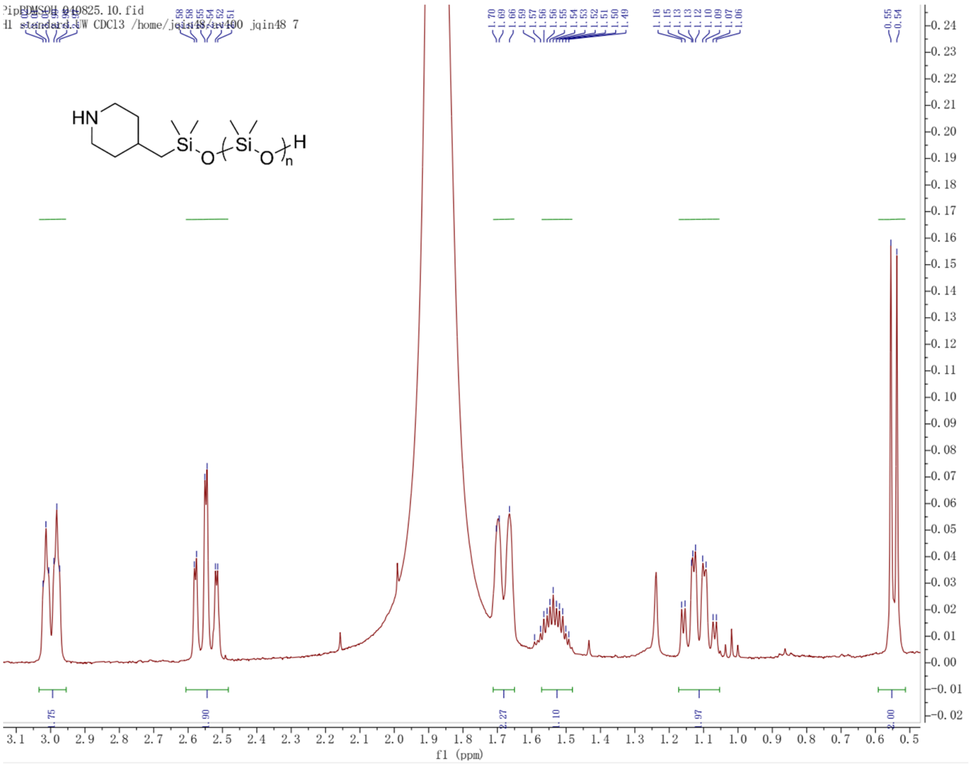

Figure S7. NMR of PiperidinePDMS magnified end group peaks

^{1}H NMR (400 MHz, $CDCl_3$) δ 3.00 (dt, *J* = 12.6, 3.3 Hz, 2H), 2.55 (td, *J* = 12.3, 2.6 Hz, 2H), 1.69 (d, 2H), 1.61 – 1.44 (m, 1H), 1.20 – 1.02 (m, 2H), 0.55 (d, *J* = 6.9 Hz, 2H), 0.11 (s, 28H), 0.08 – 0.03 (m, 574H).

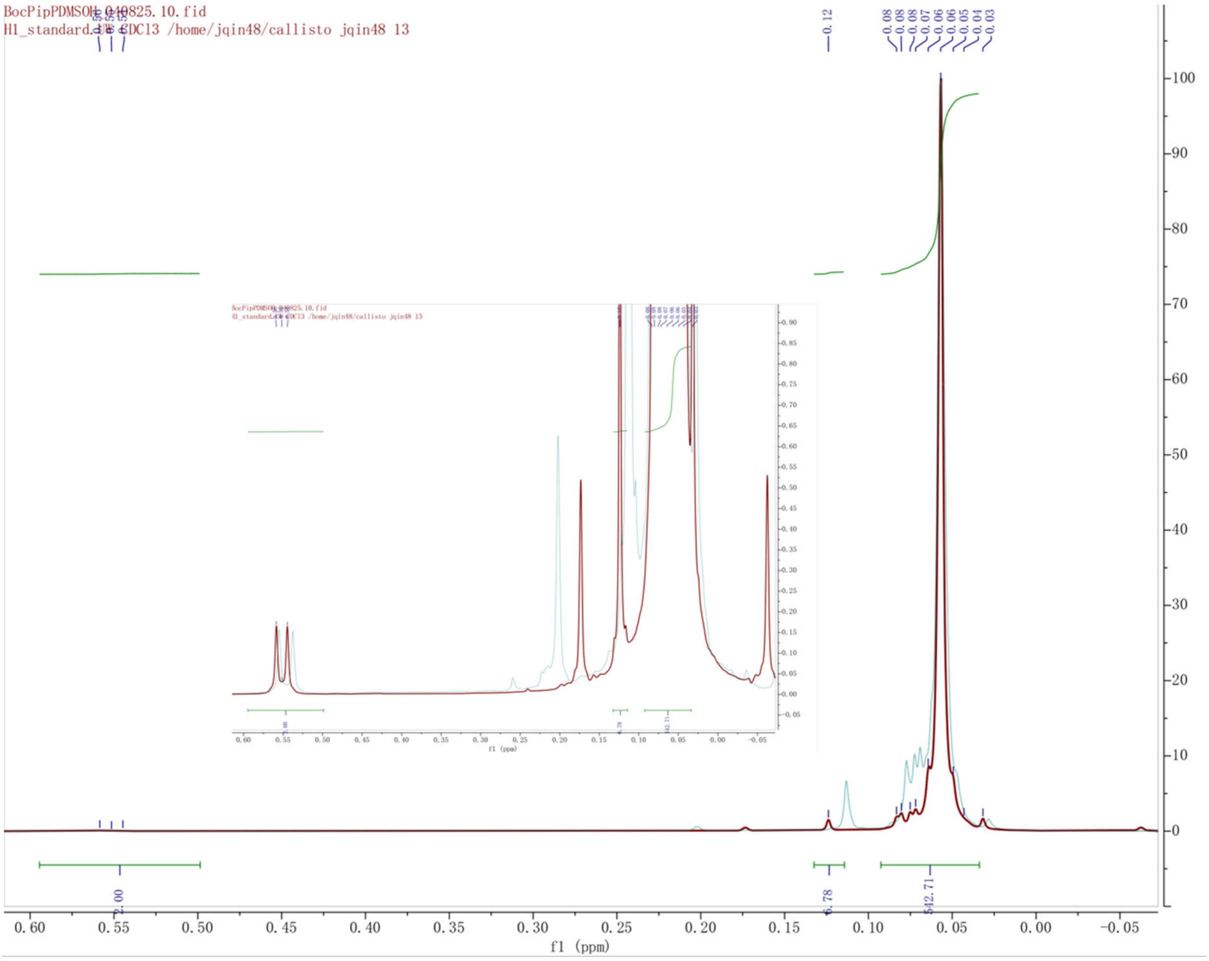

Figure S8. Relative NMR peaks of PDMS chain end and backbone before (red) and after deprotection (blue), peak at 0.55ppm belongs to $-CH_2-$ between piperidine ring and Si.

Size exclusion chromatography

ASTRA Report 052225_BocPDMSOH_P4[Sequence20250522]

ASTRA Report 052225_BocPDMSOH_P4[Sequence20250522]

WYATT TECHNOLOGY

File Properties

File Name: C:\Users\GPC computer\Documents\GPC\Sequence\052225_BocPDMSOH_P4[Sequence20250522].afe7
Created: May 22, 2025 15:02:25.583
Modified: May 23, 2025 08:47:49.698

Sample: PS-Standard1-MET
dn/dc: 0.1845 mL/g
UV Ext. Coef.: 0.000 mL/(mg cm)
Concentration: 0.000 mg/mL

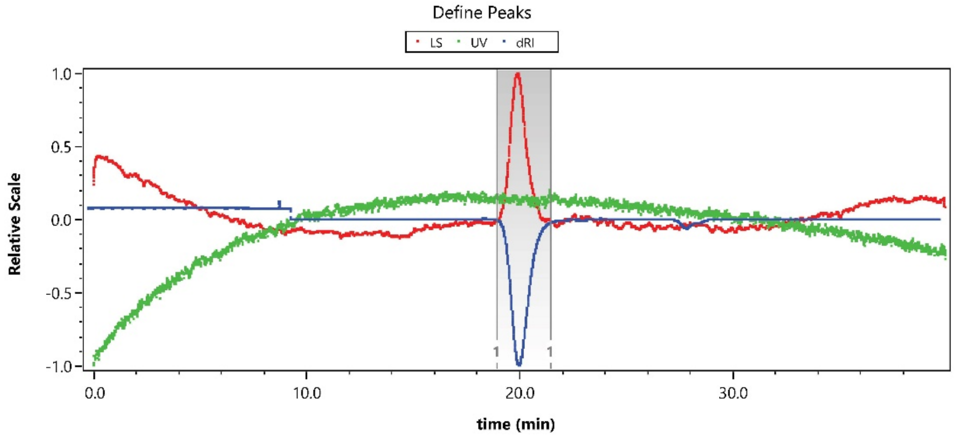

Configuration

Concentration Source: RI
Flow Rate: 1.000 mL/min

Light Scattering Instrument: miniDAWN
Cell Type: Fused Silica
Wavelength: 663.0 nm
Calibration Constant: 3.1186×10[illegible] 1/(V cm)

RI Instrument: Generic RI
Wavelength: 800.0 nm

UV Instrument: Generic UV

Solvent: THF
Refractive Index: 1.402

Processing

Collection Operator: DESKTOP-OUHTTU8\GPC computer (DESKTOP-OUHTTU8\GPC computer (GPC computer))
Collection Time: Thursday May 22, 2025 02:22:25 PM -0500
Processing Operator: DESKTOP-OUHTTU8\GPC computer (GPC computer)
Processing Time: Friday May 23, 2025 08:47:50 AM -0500

Peak settings:

**Peak Name**	Peak 1
**Peak Limits (min)**	18.946 - 21.457
**Light Scattering Model**	Zimm
**Fit Degree**	1
**dn/dc (mL/g)**	-0.0806
**A2 (mol mL/g²)**	0.000

Results Fitting Procedure:

Data	Fit Model	Degree	R^2	Extrapolation

Results

Peak Results

	Peak 1
**General (mL/(mg cm))**	
**UV Ext. Coef. (mL/(mg cm))**	0.001
**Masses**	
**Injected Mass (µg)**	105.64
**Calculated Mass (µg)**	127.11
**Mass Recovery (%)**	120.3
**Molar mass moments (g/mol)**	
**Mn**	3.362×10^{3} (±21.266%)
**Mw**	3.883×10^{3} (±14.576%)
**Polydispersity**	
**Mw/Mn**	1.155 (±25.782%)
**rms radius moments (nm)**	
**rz**	34.3 (±66.5%)

Figure S9. GPC of BocPiperidinePDMS2.6K

ASTRA Report 051825_BocPipPDMSOH_P1[051925]

WYATT TECHNOLOGY

File Properties

File Name: C:\Users\GPC computer\Documents\GPC\BKT\051825_BocPipPDMSOH_P1[051925].afe7
Created: May 19, 2025 11:10:21.489
Modified: May 20, 2025 13:09:59.785

Sample: PS-Standard1-MET
dn/dc: 0.1845 mL/g
UV Ext. Coef.: 0.000 mL/(mg cm)
Concentration: 0.000 mg/mL

Define Peaks

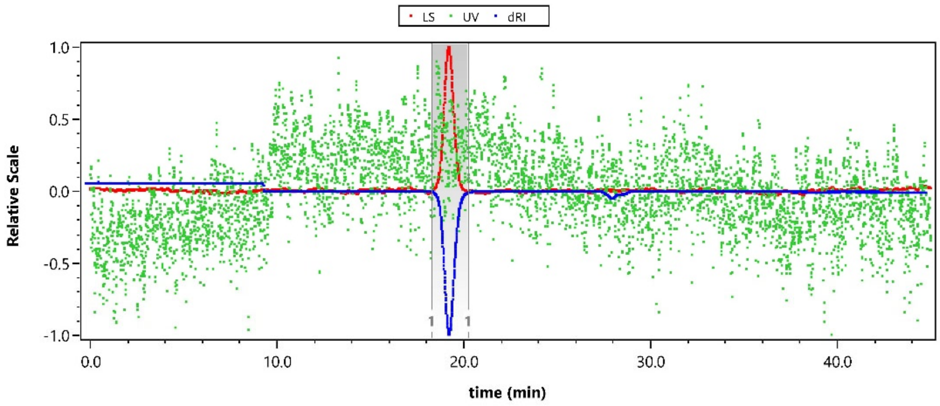

Configuration

Concentration Source: RI
Flow Rate: 1.000 mL/min

Light Scattering Instrument: miniDAWN
Cell Type: Fused Silica
Wavelength: 663.0 nm
Calibration Constant: 3.1186×10^{-5} 1/(V cm)

RI Instrument: Generic RI
Wavelength: 800.0 nm

UV Instrument: Generic UV

Solvent: THF
Refractive Index: 1.402

Processing

Collection Operator: DESKTOP-OUHTTU8\GPC computer (DESKTOP-OUHTTU8\GPC computer (GPC computer))
Collection Time: Monday May 19, 2025 10:25:21 AM -0500
Processing Operator: DESKTOP-OUHTTU8\GPC computer (GPC computer)
Processing Time: Monday May 19, 2025 12:52:49 PM -0500

Peak settings:

ASTRA Report 051825_BocPipPDMSOH_P1[051925]

**Peak Name**	Peak 1
**Peak Limits (min)**	18.249 - 20.260
**Light Scattering Model**	Zimm
**Fit Degree**	1
**dn/dc (mL/g)**	-0.0806
**A2 (mol mL/g^2)**	0.000

Results Fitting Procedure:

Data	Fit Model	Degree	R^2	Extrapolation

Results

Peak Results

	Peak 1
**General (mL/(mg cm))**	
**UV Ext. Coef. (mL/(mg cm))**	0.000
**Masses**	
**Injected Mass (µg)**	105.64
**Calculated Mass (µg)**	108.05
**Mass Recovery (%)**	102.3
**Molar mass moments (g/mol)**	
**Mn**	7.289×10^3 (±3.066%)
**Mw**	7.551×10^3 (±1.907%)
**Polydispersity**	
**Mw/Mn**	1.036 (±3.611%)
**rms radius moments (nm)**	
**rz**	n/a

ASTRA 7.3.2.21 5/20/2025 1:11:47 PM 2 of 2

Figure S10. GPC of BocPiperidinePDMS5.8K

ASTRA Report 051825_BocPipPDMSOH_P3[051925]

File Properties

File Name: C:\Users\GPC computer\Documents\GPC\BKT\051825_BocPipPDMSOH_P3[051925].afe7
Created: May 19, 2025 12:56:59.298
Modified: May 20, 2025 13:14:21.847

Sample: 051825_BocPipPDMSOH_P3
dn/dc: 0.1845 mL/g
UV Ext. Coef.: 0.000 mL/(mg cm)
Concentration: 5.363 mg/mL

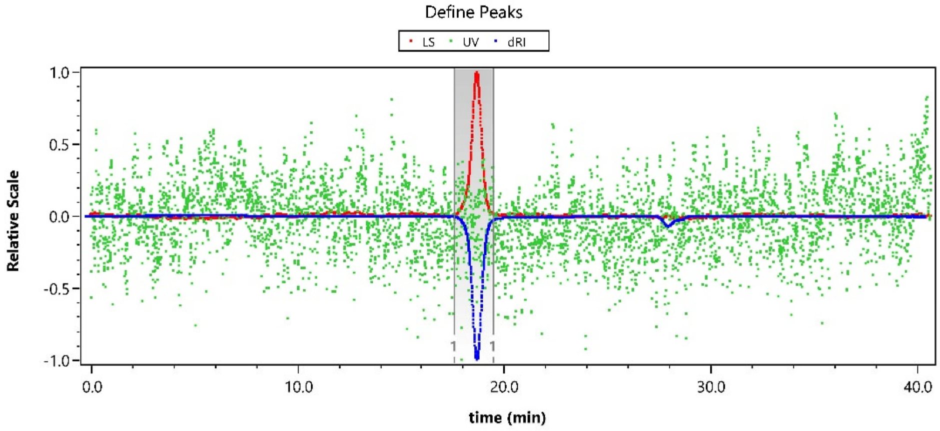

Configuration

Concentration Source: RI
Flow Rate: 1.000 mL/min

Light Scattering Instrument: miniDAWN
Cell Type: Fused Silica
Wavelength: 663.0 nm
Calibration Constant: 3.1186×10^{-5} 1/(V cm)

RI Instrument: Generic RI
Wavelength: 800.0 nm

UV Instrument: Generic UV

Solvent: THF
Refractive Index: 1.402

Processing

Collection Operator: DESKTOP-OUHTTU8\GPC computer (DESKTOP-OUHTTU8\GPC computer (GPC computer))
Collection Time: Monday May 19, 2025 12:16:20 PM -0500
Processing Operator: DESKTOP-OUHTTU8\GPC computer (GPC computer)
Processing Time: Monday May 19, 2025 12:58:09 PM -0500

Peak settings:

**Peak Name**	Peak 1
**Peak Limits (min)**	17.549 - 19.454
**Light Scattering Model**	Zimm
**Fit Degree**	1
**dn/dc (mL/g)**	-0.0806
**A2 (mol mL/g²)**	0.000

Results Fitting Procedure:

Data	Fit Model	Degree	R^2	Extrapolation

Results

Peak Results

	Peak 1
**General (mL/(mg cm))**	
**UV Ext. Coef. (mL/(mg cm))**	-0.000
**Masses**	
**Injected Mass (µg)**	105.64
**Calculated Mass (µg)**	66.30
**Mass Recovery (%)**	62.8
**Molar mass moments (g/mol)**	
**Mn**	1.371×10^4 (±1.866%)
**Mw**	1.405×10^4 (±2.130%)
**Polydispersity**	
**Mw/Mn**	1.025 (±2.831%)
**rms radius moments (nm)**	
**rz**	19.8 (±26.8%)

Figure S11. GPC of BocPiperidinePDMS10.5K

ASTRA Report untitled002[Sequence20250619]

File Properties

File Name: C:\Users\GPC computer\Documents\GPC\Sequence\untitled002[Sequence20250619].afe7
Created: June 19, 2025 14:53:59.982
Modified: June 19, 2025 14:55:13.434

Sample:

dn/dc: 0.1845 mL/g
UV Ext. Coef.: 0.000 mL/(mg cm)
Concentration: 0.000 mg/mL

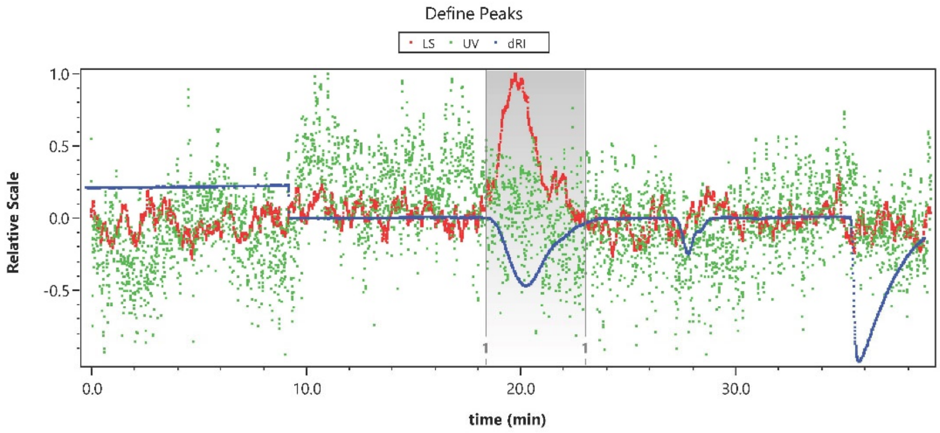

Configuration

Concentration Source: RI
Flow Rate: 1.000 mL/min

Light Scattering Instrument: miniDAWN

Cell Type: Fused Silica
Wavelength: 663.0 nm
Calibration Constant: 3.1186×10^{-5} 1/(V cm)

RI Instrument: Generic RI

Wavelength: 800.0 nm

UV Instrument: Generic UV

Solvent: THF

Refractive Index: 1.402

Processing

Collection Operator: DESKTOP-OUHTTU8\GPC computer (DESKTOP-OUHTTU8\GPC computer (GPC computer))
Collection Time: Thursday June 19, 2025 02:14:53 PM -0500
Processing Operator: DESKTOP-OUHTTU8\GPC computer (GPC computer)
Processing Time: Thursday June 19, 2025 02:55:13 PM -0500

Peak settings:

**Peak Name**	Peak 1
**Peak Limits (min)**	18.404 - 23.004
**Light Scattering Model**	Zimm
**Fit Degree**	1
**dn/dc (mL/g)**	-0.0806
**A2 (mol mL/g²)**	0.000

Results Fitting Procedure:

Data	Fit Model	Degree	R^2	Extrapolation

Results

Peak Results

	Peak 1
**General (mL/(mg cm))**	
**UV Ext. Coef. (mL/(mg cm))**	-0.000
**Masses**	
**Injected Mass (µg)**	105.64
**Calculated Mass (µg)**	46.02
**Mass Recovery (%)**	43.6
**Molar mass moments (g/mol)**	
**Mn**	2.829×10^3 (±33.963%)
**Mw**	3.638×10^3 (±30.130%)
**Polydispersity**	
**Mw/Mn**	1.286 (±45.402%)
**rms radius moments (nm)**	
**rz**	17.3 (±364.9%)

Figure S12. GPC of PiperidinePDMS2.6K. Peak at 28min belongs to the oligomer from the hydrolysis.

ASTRA Report 051825_PipPDMSOH_P1[Sequence20250519]

WYATT TECHNOLOGY

File Properties

File Name: C:\Users\GPC computer\Documents\GPC\Sequence\051825_PipPDMSOH_P1[Sequence20250519].afe7
Created: May 19, 2025 18:03:27.021
Modified: May 20, 2025 13:03:41.651

Sample: PS-Standard1-MET
dn/dc: 0.1845 mL/g
UV Ext. Coef.: 0.000 mL/(mg cm)
Concentration: 0.000 mg/mL

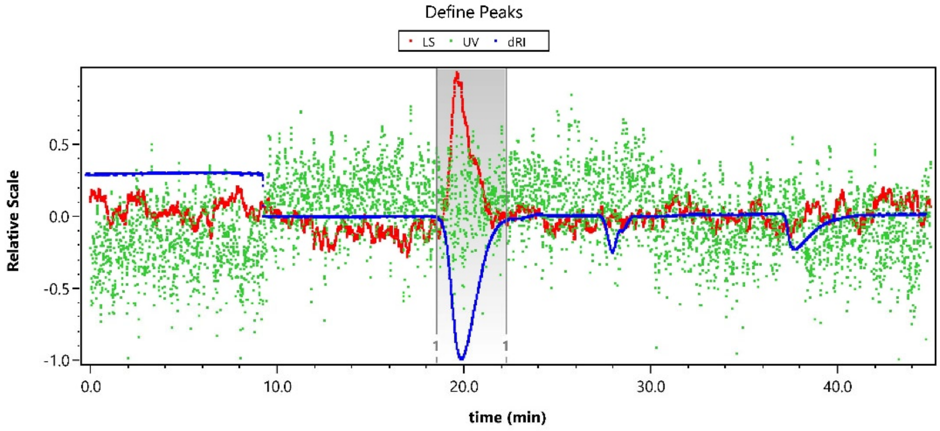

Configuration

Concentration Source: RI
Flow Rate: 1.000 mL/min

Light Scattering Instrument: miniDAWN
Cell Type: Fused Silica
Wavelength: 663.0 nm
Calibration Constant: 3.1186×10^{-5} 1/(V cm)

RI Instrument: Generic RI
Wavelength: 800.0 nm

UV Instrument: Generic UV

Solvent: THF
Refractive Index: 1.402

Processing

Collection Operator: DESKTOP-OUHTTU8\GPC computer (DESKTOP-OUHTTU8\GPC computer (GPC computer))
Collection Time: Monday May 19, 2025 05:18:26 PM -0500
Processing Operator: DESKTOP-OUHTTU8\GPC computer (GPC computer)
Processing Time: Tuesday May 20, 2025 01:03:42 PM -0500

Peak settings:

**Peak Name**	Peak 1
**Peak Limits (min)**	18.543 - 22.274
**Light Scattering Model**	Zimm
**Fit Degree**	1
**dn/dc (mL/g)**	-0.0806
**A2 (mol mL/g²)**	0.000

Results Fitting Procedure:

Data	Fit Model	Degree	R^2	Extrapolation

Results

Peak Results

	Peak 1
**General (mL/(mg cm))**	
**UV Ext. Coef. (mL/(mg cm))**	0.000
**Masses**	
**Injected Mass (µg)**	105.64
**Calculated Mass (µg)**	53.67
**Mass Recovery (%)**	50.8
**Molar mass moments (g/mol)**	
**Mn**	3.492×10^3 (±20.072%)
**Mw**	3.813×10^3 (±16.431%)
**Polydispersity**	
**Mw/Mn**	1.092 (±25.940%)
**rms radius moments (nm)**	
**rz**	n/a

Figure S13. GPC of PiperidinePDMS5.8K

ASTRA Report PipPDMSTMS_061225_P4[Sequence20250612]

File Properties

File Name: C:\Users\GPC computer\Documents\GPC\Sequence\PipPDMSTMS_061225_P4[Sequence20250612].afe7
Created: June 12, 2025 14:25:41.277
Modified: June 12, 2025 14:29:28.966

Sample: PS-Standard1-MET
dn/dc: 0.1845 mL/g
UV Ext. Coef.: 0.000 mL/(mg cm)
Concentration: 0.000 mg/mL

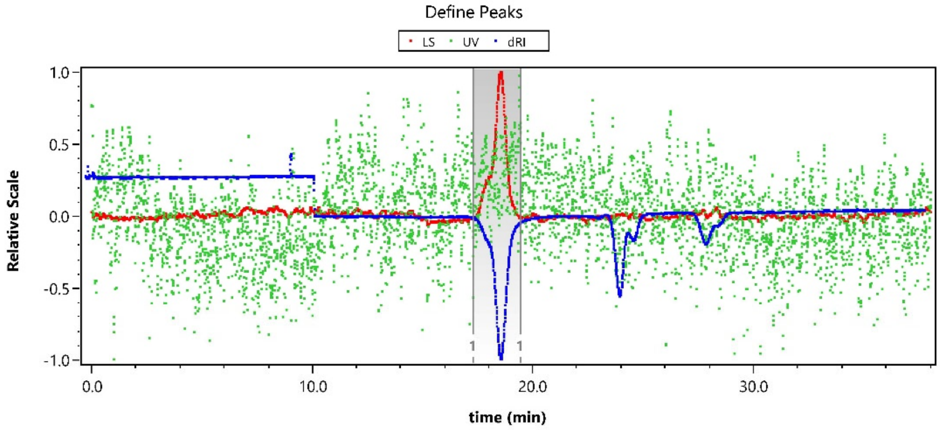

Configuration

Concentration Source: RI
Flow Rate: 1.000 mL/min

Light Scattering Instrument: miniDAWN
Cell Type: Fused Silica
Wavelength: 663.0 nm
Calibration Constant: 3.1186×10^{-5} 1/(V cm)

RI Instrument: Generic RI
Wavelength: 800.0 nm

UV Instrument: Generic UV

Solvent: THF
Refractive Index: 1.402

Processing

Collection Operator: DESKTOP-OUHTTU8\GPC computer (DESKTOP-OUHTTU8\GPC computer (GPC computer))
Collection Time: Thursday June 12, 2025 01:47:36 PM -0500
Processing Operator: DESKTOP-OUHTTU8\GPC computer (GPC computer)
Processing Time: Thursday June 12, 2025 02:29:29 PM -0500

Peak settings:

**Peak Name**	Peak 1
**Peak Limits (min)**	17.288 - 19.459
**Light Scattering Model**	Zimm
**Fit Degree**	1
**dn/dc (mL/g)**	-0.0806
**A2 (mol mL/g^2)**	0.000

Results Fitting Procedure:

Data	Fit Model	Degree	R^2	Extrapolation

Results

Peak Results

	Peak 1
**General (mL/(mg cm))**	
UV Ext. Coef. (mL/(mg cm))	0.000
**Masses**	
Injected Mass (μg)	105.64
Calculated Mass (μg)	24.37
Mass Recovery (%)	23.1
**Molar mass moments (g/mol)**	
Mn	1.355×10^4 (±9.796%)
Mw	1.466×10^4 (±8.341%)
**Polydispersity**	
Mw/Mn	1.082 (±12.866%)
**rms radius moments (nm)**	
rz	n/a

Figure S14. GPC of PiperidinePDMS10.5K. Peak at 24min belongs to the petroleum ether used for transferring PDMS.

Structural Characterization

Small Angle X-Ray Scattering (SAXS) of Homopolymers

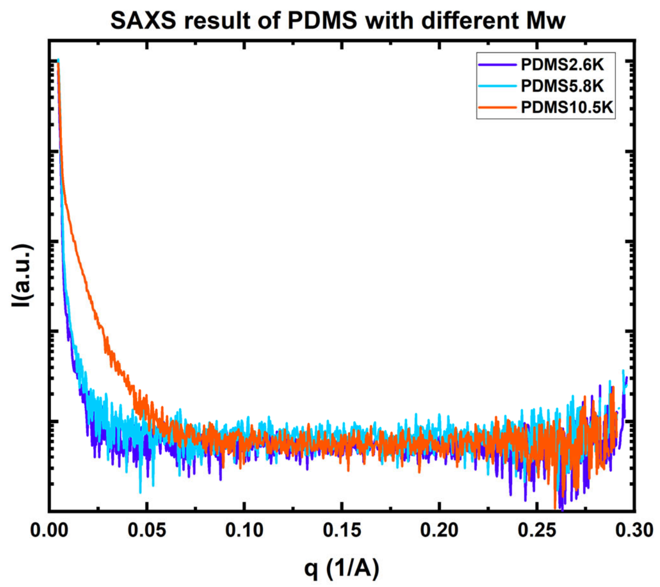

Figure S15. SAXS result of Pip-PDMS Homopolymer at room temperature

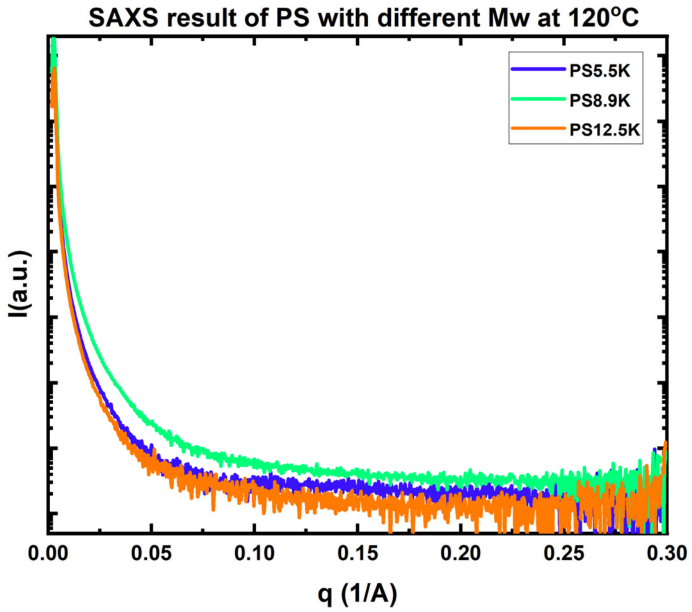

Figure S16. SAXS result of PS Homopolymer at 120 °C

FTIR and NMR characterization of blends

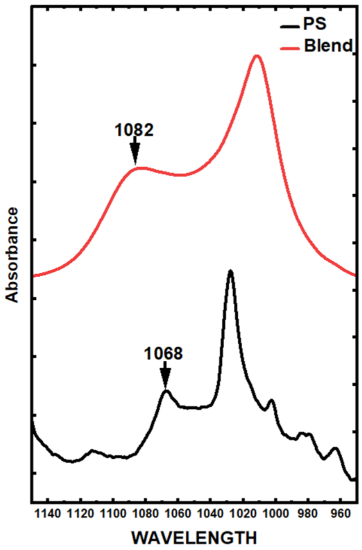

Figure S17. FTIR spectra of PS and its stoichiometric blend

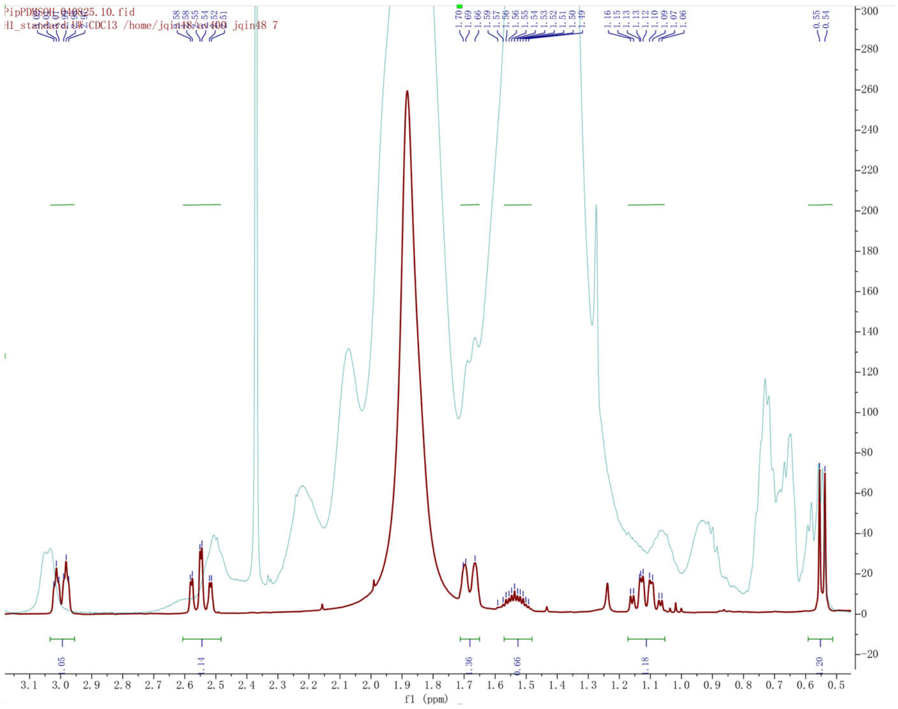

Figure S18. NMR peaks of PDMS chain end area in PDMS (red) and Blends (blue)

The formation of -SO_3H/-Pip ion pairs association was confirmed via FTIR spectra and ^1H NMR. The PS and blends' FTIR peaks in the range between 1150 to 950 cm^{-1} before and after mixing is consistent with previous report, see Figure S17. In the range from 1150 to 950 cm^{-1}, the peak at 1068 cm^{-1} could be assigned to the SO_3^- group at the end of PS chain. After mixing with PDMS, the peak shifted and

became broad due to neutralization. The peak shift is fully consistent with the previous report of PS/PDMS system with $-SO_3H/-NH_2$ end groups. The NMR peak change of PDMS's piperidine end group also shows neutralization happens, Figure S18. The peak at 0.55 ppm belongs to the junction part ($-CH_2-$ between piperidine ring and Si), the peaks between 2.5-2.6ppm and 3.0-3.1 ppm belongs to the piperidine ring. After mixing, the significant peak shape change and slight chemical shift change shows the chemical environment change, which could be assigned to the neutralization of -NH-.

Small Angle X-Ray Scattering (SAXS) result of PDMS/PS Blends

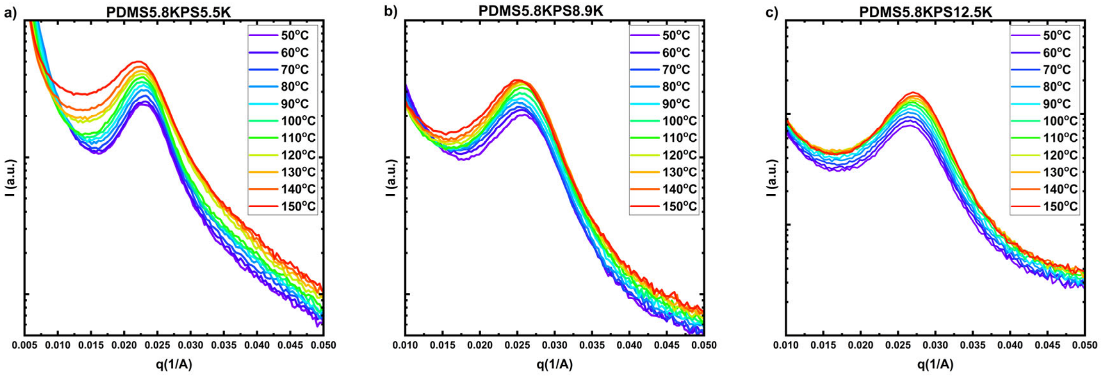

Figure S19. SAXS result of PDMS5.8K equimolar blend with series PS at different temperatures a)PS5.5K, b)PS8.9K, c)PS12.5K

Unlike the PDMS2.6K series blend, the nanostructures exhibited by the PDMS5.8K series are significantly more stable with temperature, and we observe the opposite behavior with respect to molecular weight. For these blends, higher molecular weight PS results in blends with increased temperature stability, and the FWHM of the PDMS5.8KPS8.9K and PDMS5.8KPS12.5K blends are constant in the temperature window. For the lowest molecular weight blend, PDMS5.8KPS5.5K, the scattering peak broadens and shifts at T > 110 °C. These observed differences in behavior with respect to temperature are likely due to differences in the volume fraction of association in each blend.

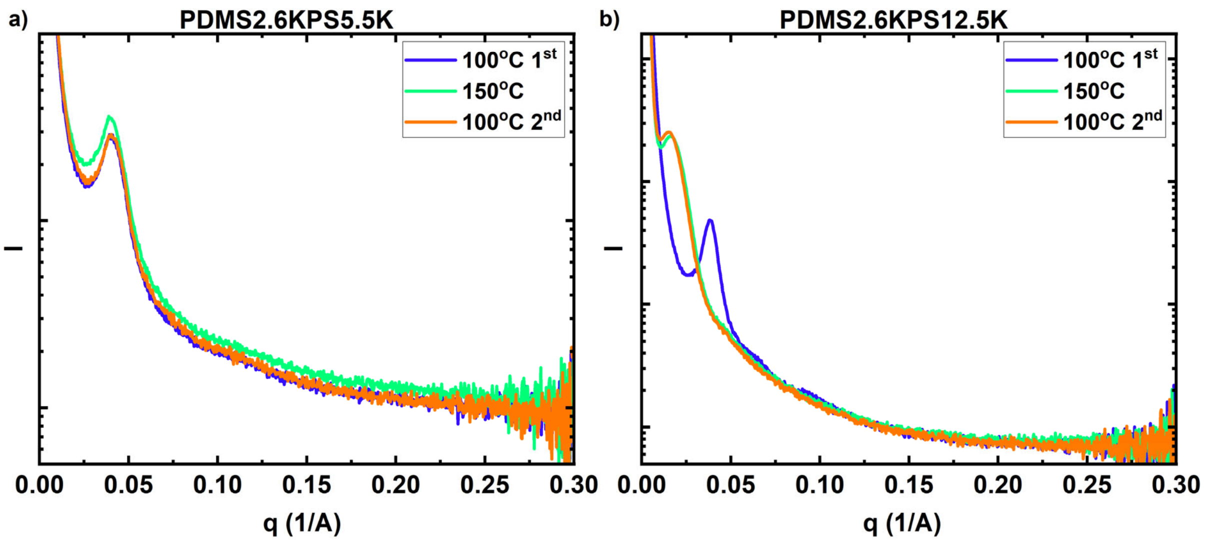

Figure S20. SAXS profile at 100 °C before (blue) and after (orange) annealing at 150 °C (green) of a) PDMS2.6KPS5.5K, b) PDMS2.6KPS12.5K

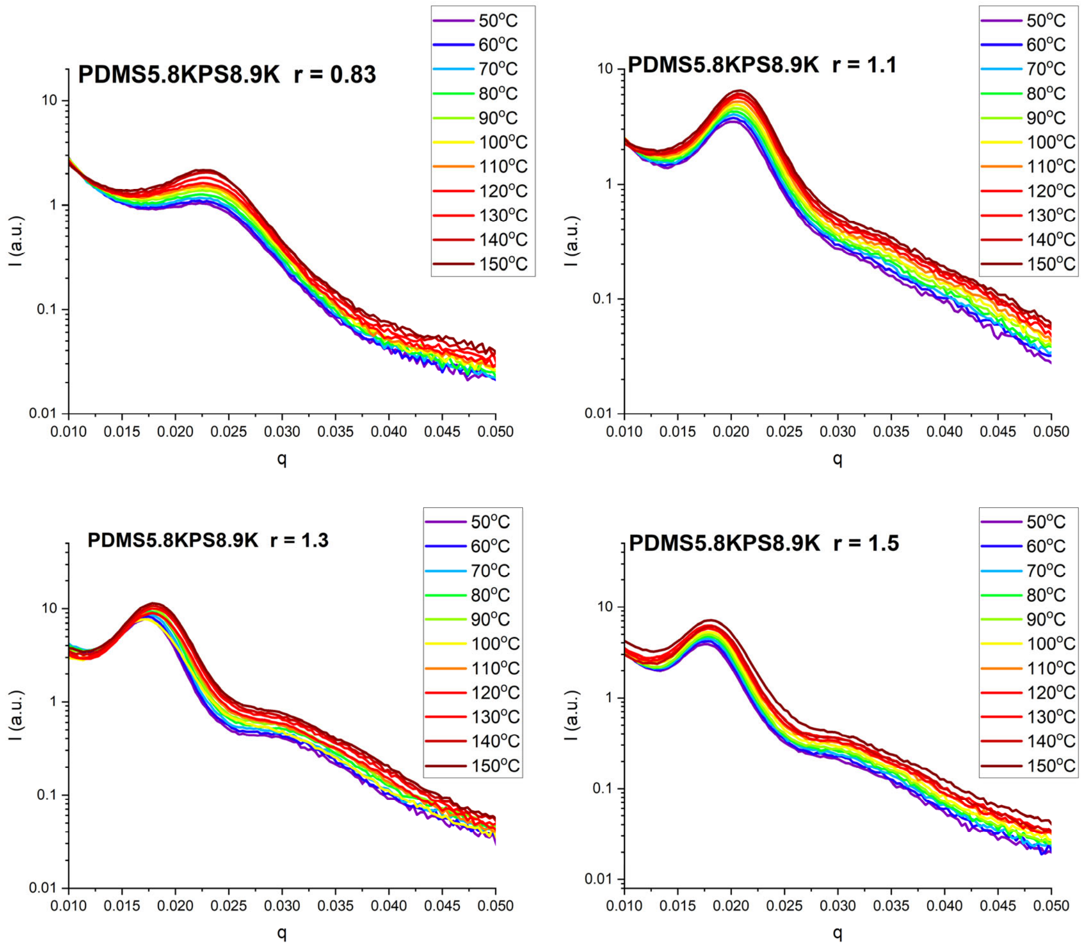

Figure S21. SAXS result of PDMS5.8KPS8.9K blend with different molar ratio at different temperatures

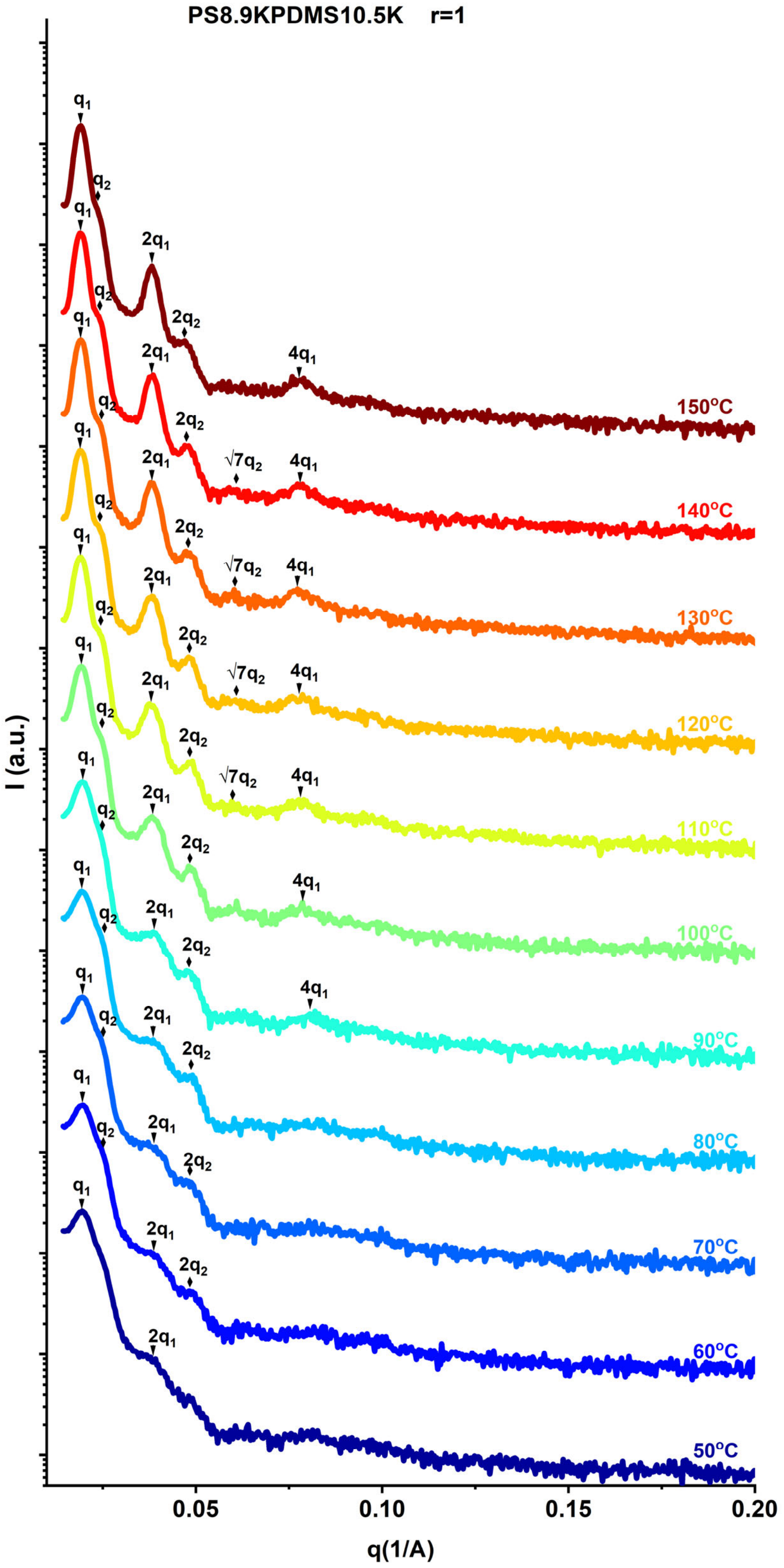

Figure S22. SAXS result of PDMS10.5KPS8.9K blends at different temperatures for $r = 1$

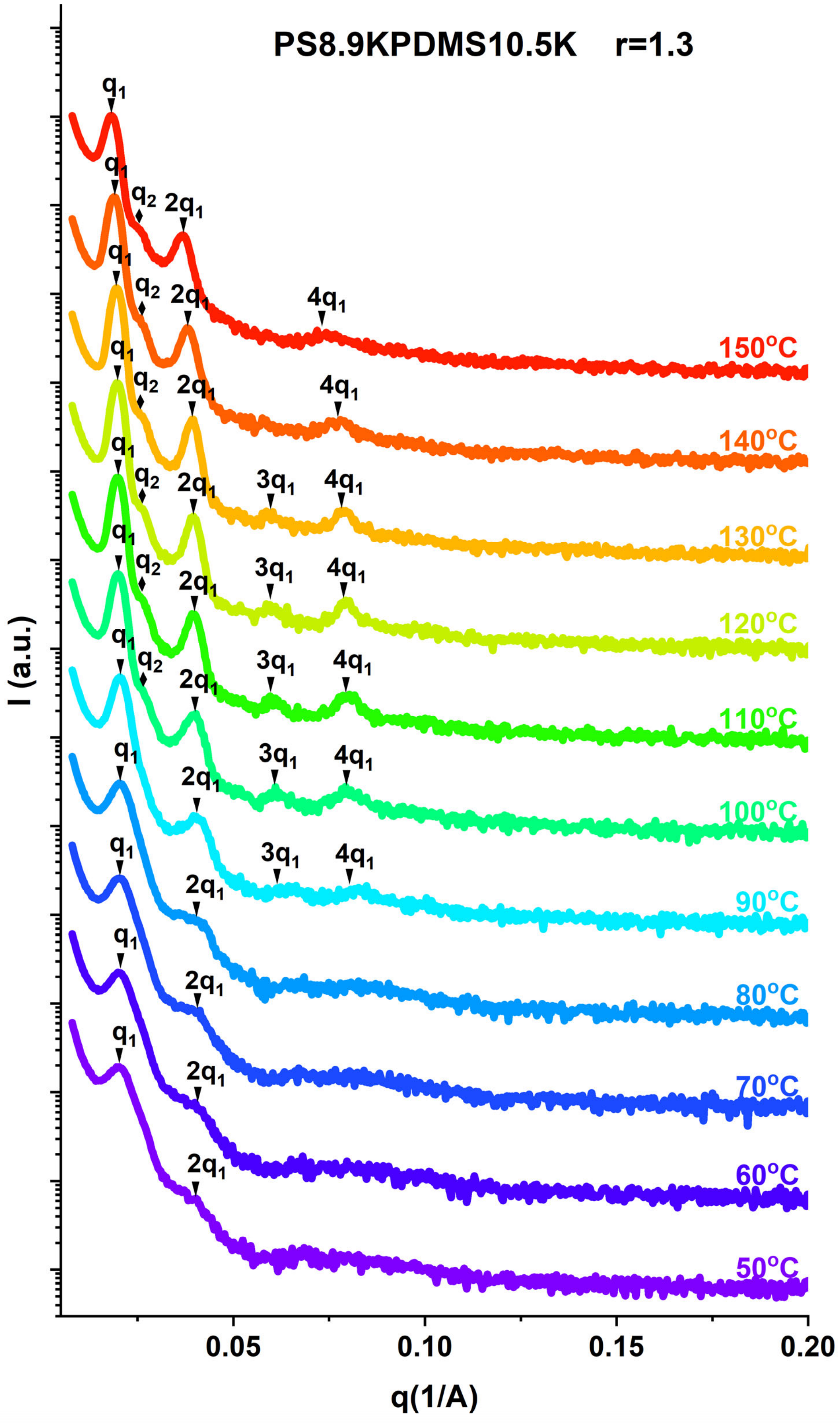

Figure S23. SAXS result of PDMS10.5KPS8.9K blends at different temperatures for $r = 1.3$

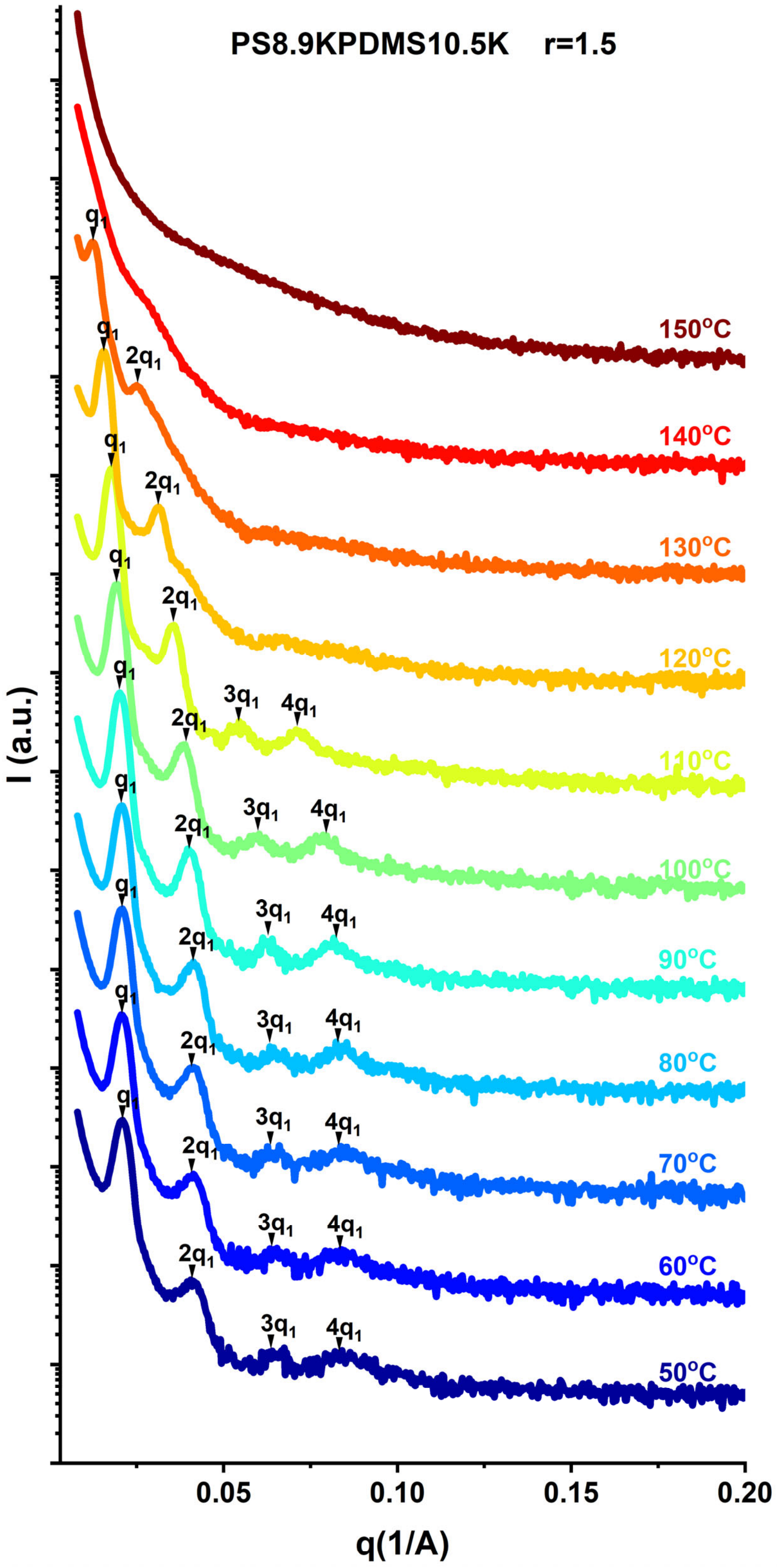

Figure S24. SAXS result of PDMS10.5KPS8.9K blends at different temperature for r = 1.5

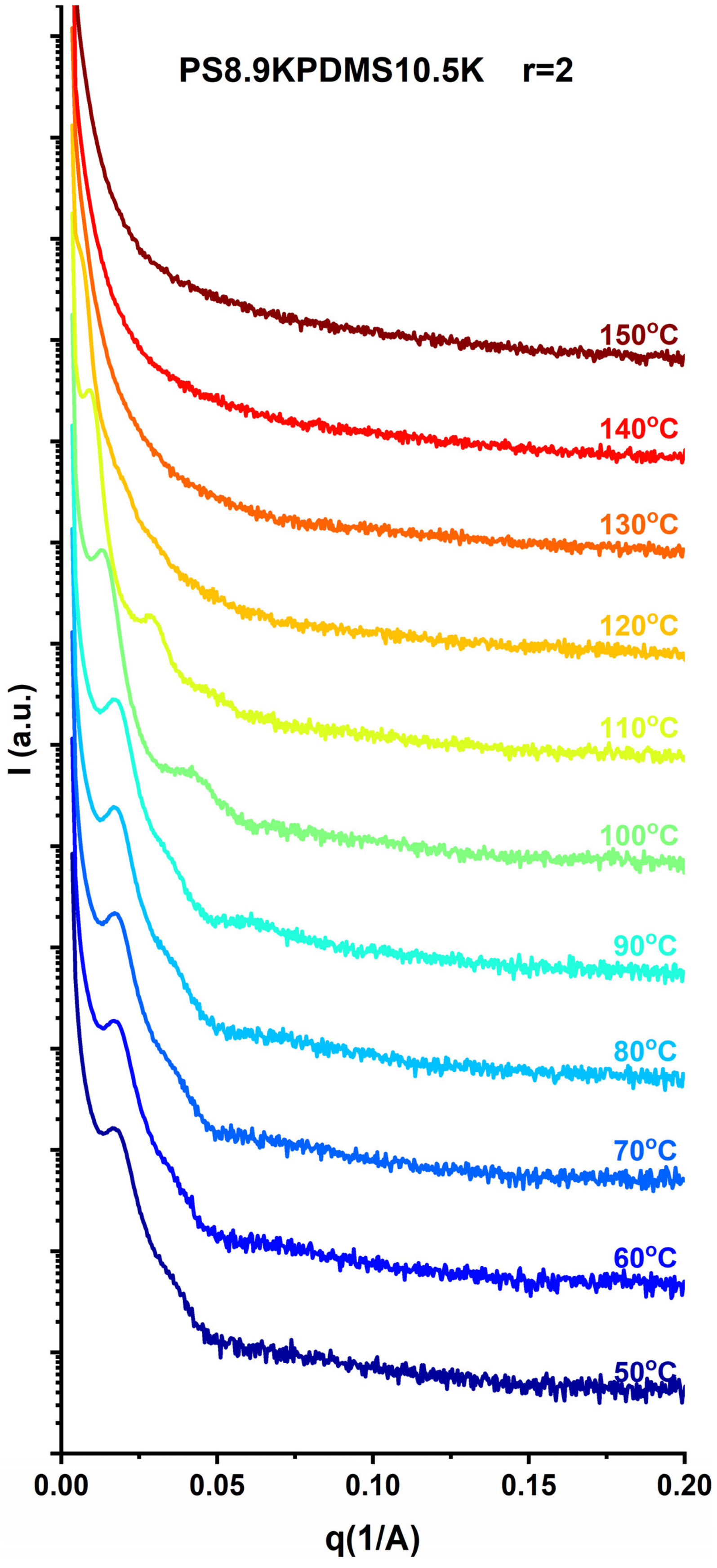

Figure S25. SAXS result of PDMS10.5KPS8.9K blends at different temperature for $r = 2$

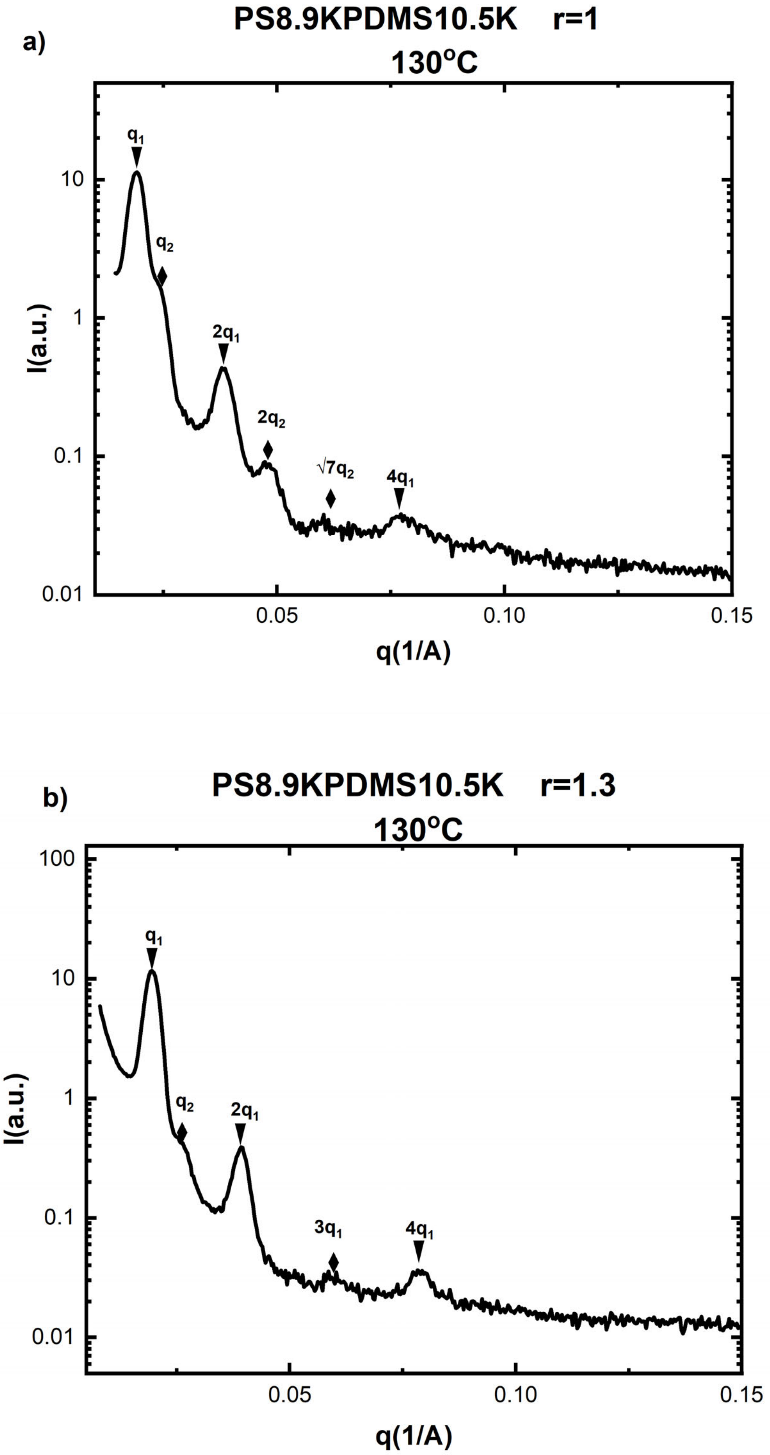

Figure S26. Peak position of PDMS10.5KPS8.9K a) $r = 1$ at 130 °C; b) $r = 1.3$ at 130 °C: Peak position of lamellae structure (triangle) and cylinder structure (square)

Table S1. Peak position of PDMS10.5KPS8.9K equimolar blend and peaks derived from different structures

Peaks from SAXS measurement										
Peak order	q_1^*	Predicted Peaks				q_2^*	Predicted Peaks			
	1/A	Sphere	Cylinder	Lamellar	Gyroid	1/A	Sphere	Cylinder	Lamellar	Gyroid
1	0.0191	0.0191	0.0191	0.0191	0.0191	0.0233	0.0233	0.0233	0.0233	0.0233
2	0.0382	0.0270	0.0331	0.0382	0.0468	0.0466	0.0330	0.0404	0.0466	0.0571
3	0.0770	0.0331	0.0382	0.0573	0.0540	0.0610	0.0404	0.0466	0.0699	0.0659
4		0.0382	0.0505	0.0764	0.0715		0.0466	0.0616	0.0932	0.0872

SAXS Fitting Result

Full Width at Half Maximum of PDMSPS Blends

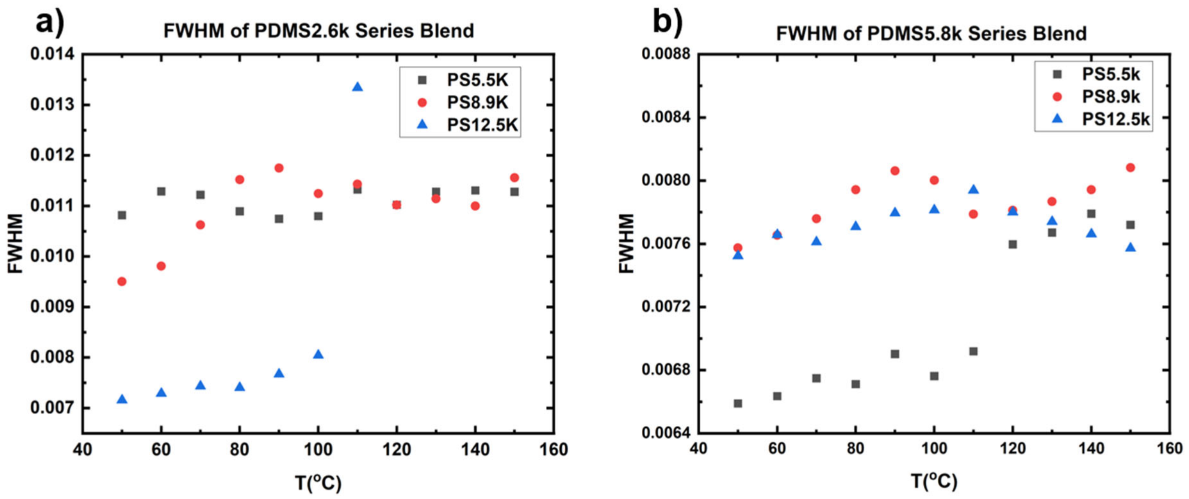

Figure S27. FWHM of equimolar blend with PS 5.5K (black), 8.9K (red) and 12.5K.(blue) with PDMS a) PDMS2.6K; b) PDMS5.8K at different temperatures.

Porod invairant of PDMSPS blends

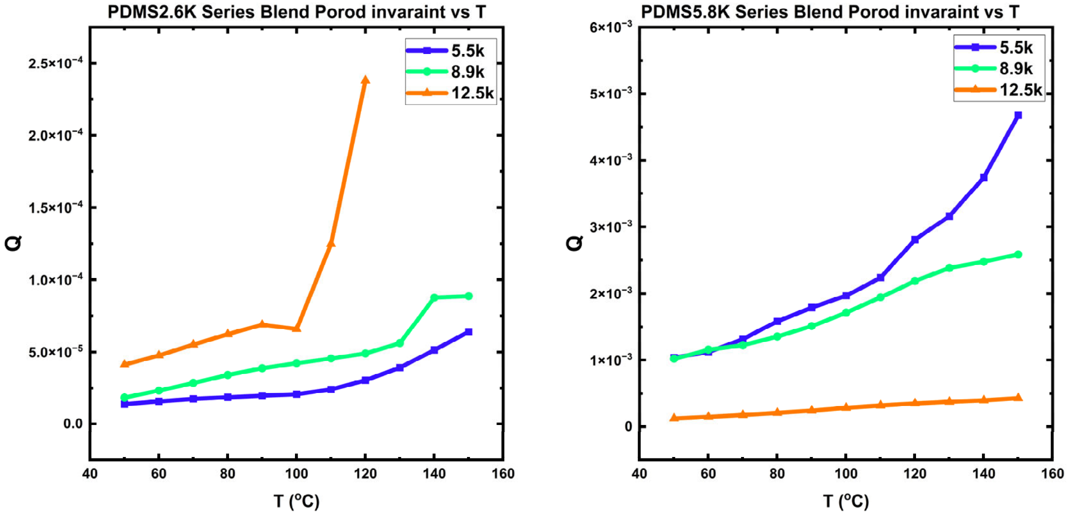

Figure S28. Porod invariant of PDMS2.6K and PDMS5.8K equimolar blend with PS 5.5K (blue), 8.9K (green) and 12.5K (orange) at different temperatures.

Total Derivation of Porod Invariant vs T

As T increases, the maximum intensity of the peak increases, a general feature of this system that has been previously reported. The FWHM doesn't show a large fluctuation, which means there isn't a significant microstructure change. The intensity here correlates with the Porod invariant, which remains unaffected by the phases' size, shapes, interactions, or arrangements. It reflects the density contrast. The function of Porod invariant

$$Q^* = \int_0^\infty q^2 I(q) dq \propto \phi_1 \phi_2 (\rho_1 - \rho_2)^2 \quad (1)$$

Due to a lack of understanding of the microstructure, the I vs q at low q and high q areas cannot be predicted, which means the total invariant is not available. Therefore, a suitable range is selected to calculate its relative change. Considering the density change due to thermo-expansion, the derivative of the $Q*$ could be in the following form

$$\frac{Q_{T_2}}{Q_{T_1}} = 1 + \left[(\alpha_1 - \alpha_2)(\phi_1 - \phi_2) + 2\left(\frac{\alpha_2 \rho_2 - \alpha_1 \rho_1}{\rho_1 - \rho_2}\right) \right] \Delta T \quad (2)$$

Here, the relative Porod invariant change could be calculated based on the temperature difference. The calculation shows that the Porod invariant change due to thermo-expansion with a 100 °C difference is 15%, while the actual value change is 60%, **Figure S16**. This indicates that the phase becomes purer after increasing the temperature. This change is reversible, as confirmed by checking the I versus q before and after reaching 150 °C at the same temperature.

Random Phase Approximation (RPA) Fitting Result

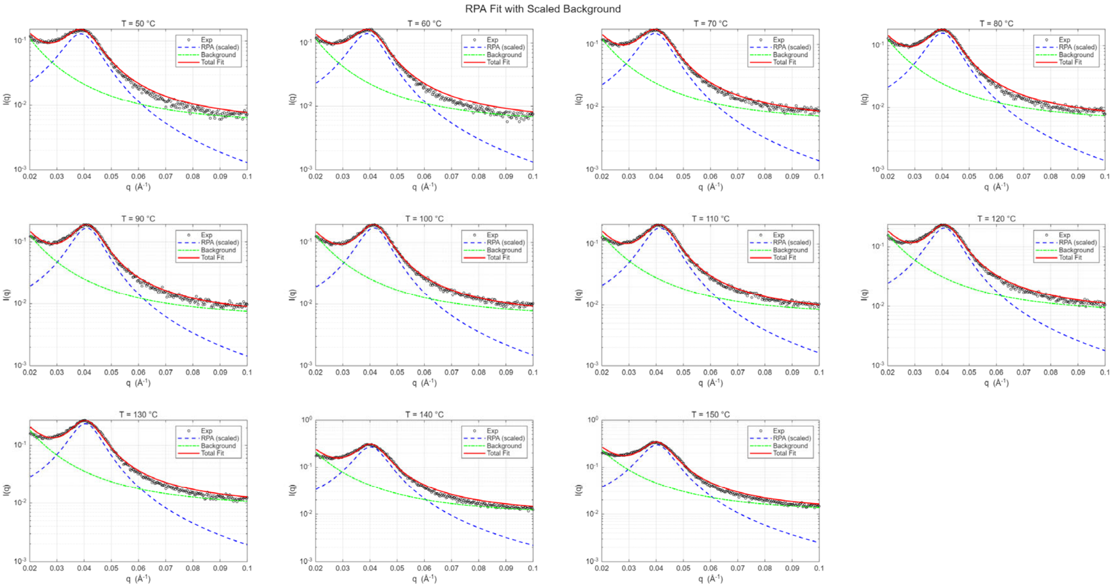

Figure S29. RPA fitting result of PDMS2.6KPS5.5K at different temperatures.

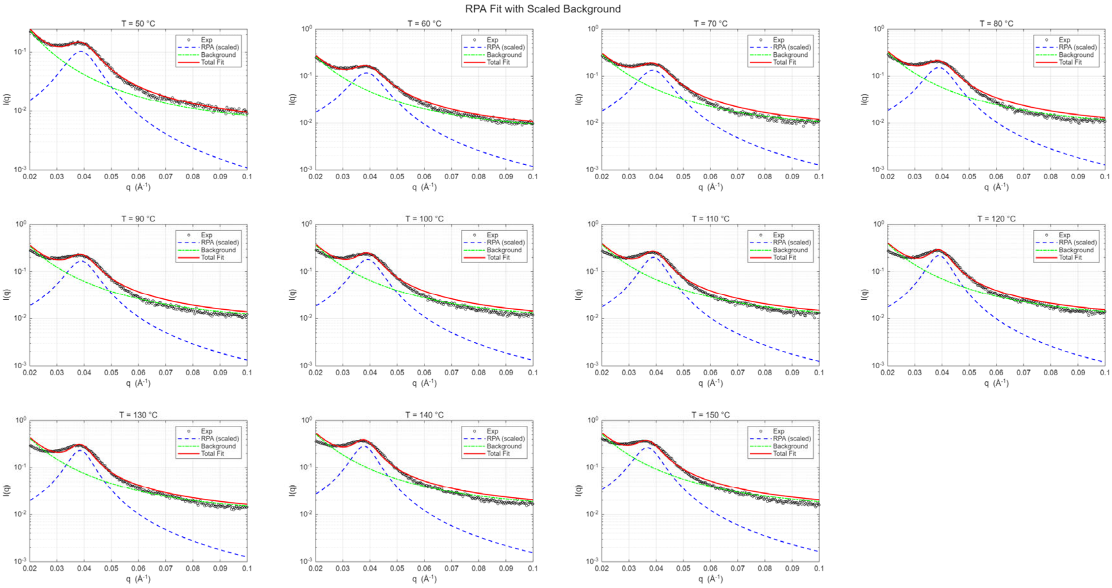

Figure S30. RPA fitting result of PDMS2.6KPS8.9K at different temperatures.

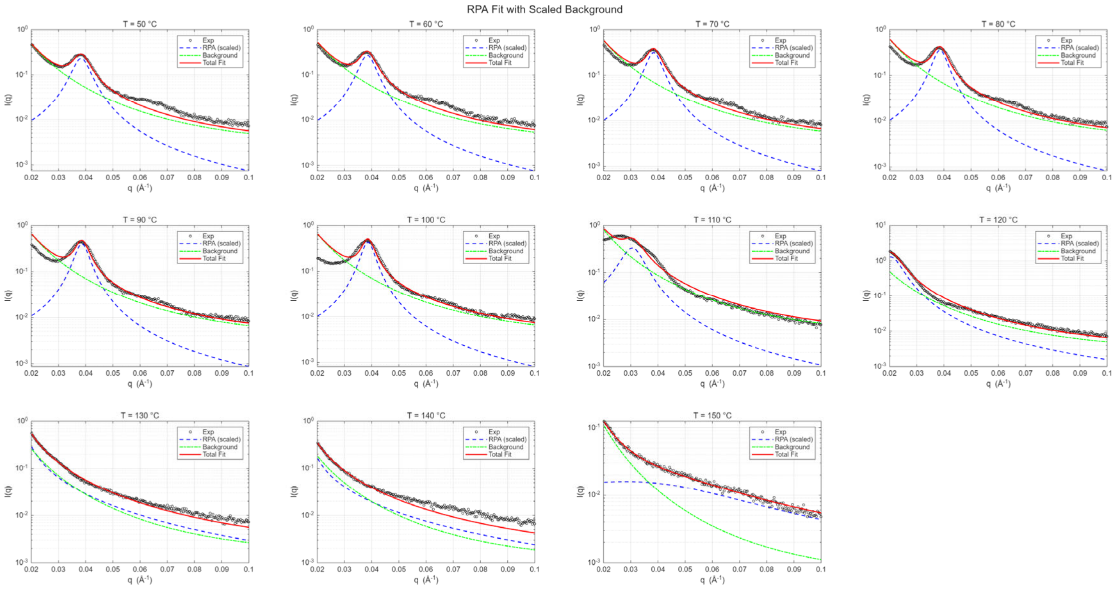

Figure S31. RPA fitting results of PDMS2.6KPS12.5K at different temperatures.

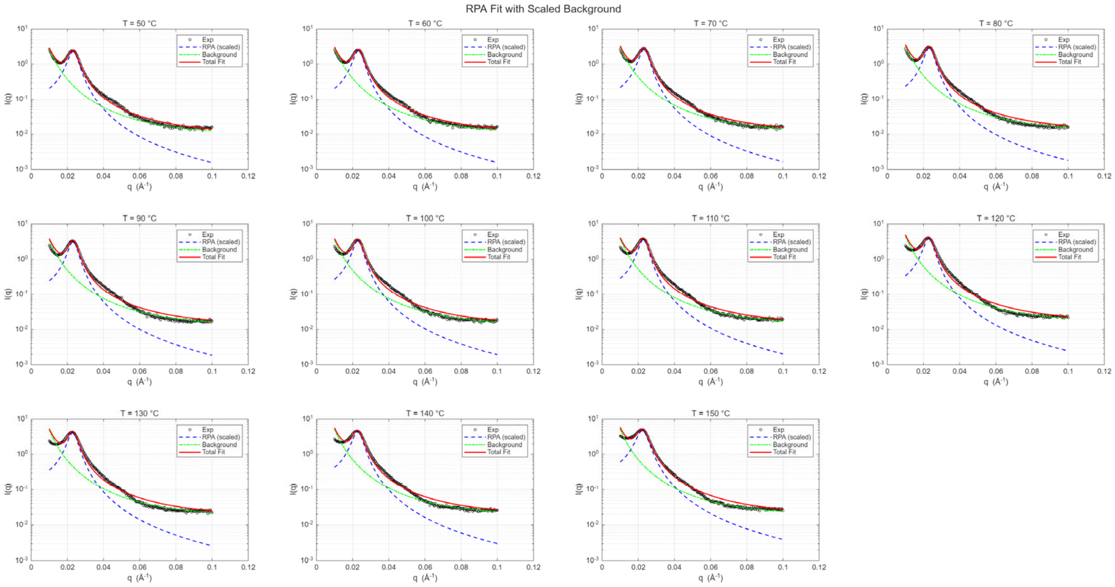

Figure S32. RPA fitting result of PDMS5.8KPS5.5K at different temperatures.

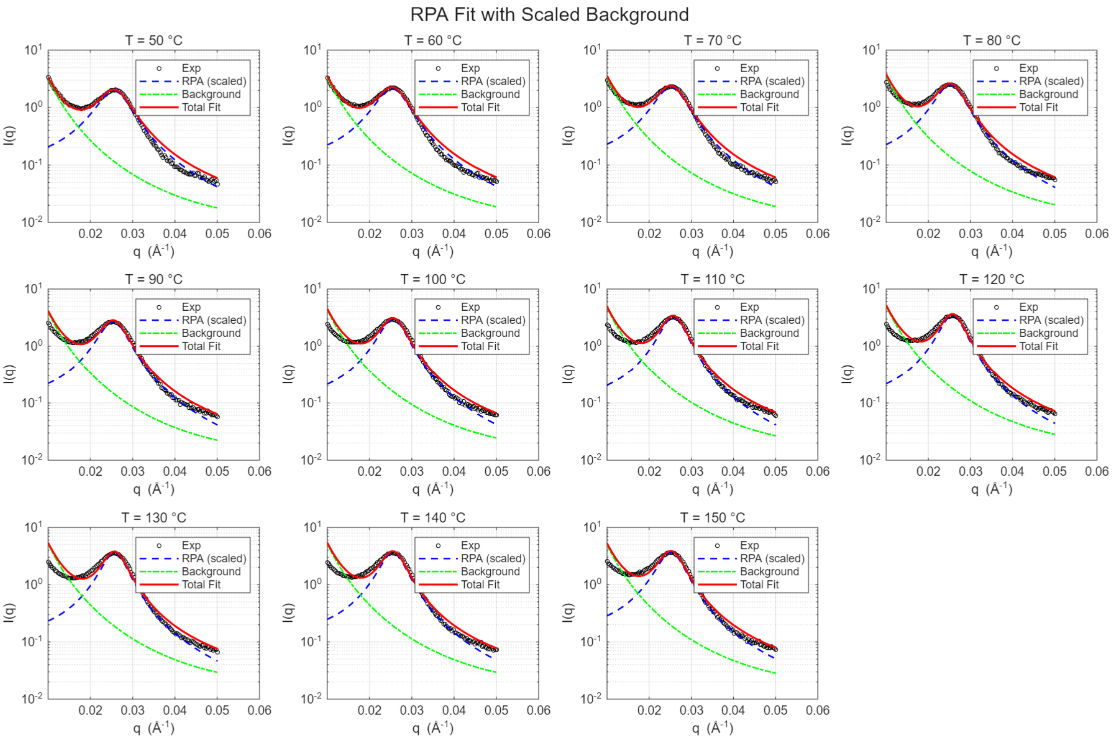

Figure S33. RPA fitting result of PDMS5.8KPS8.9K at different temperatures.

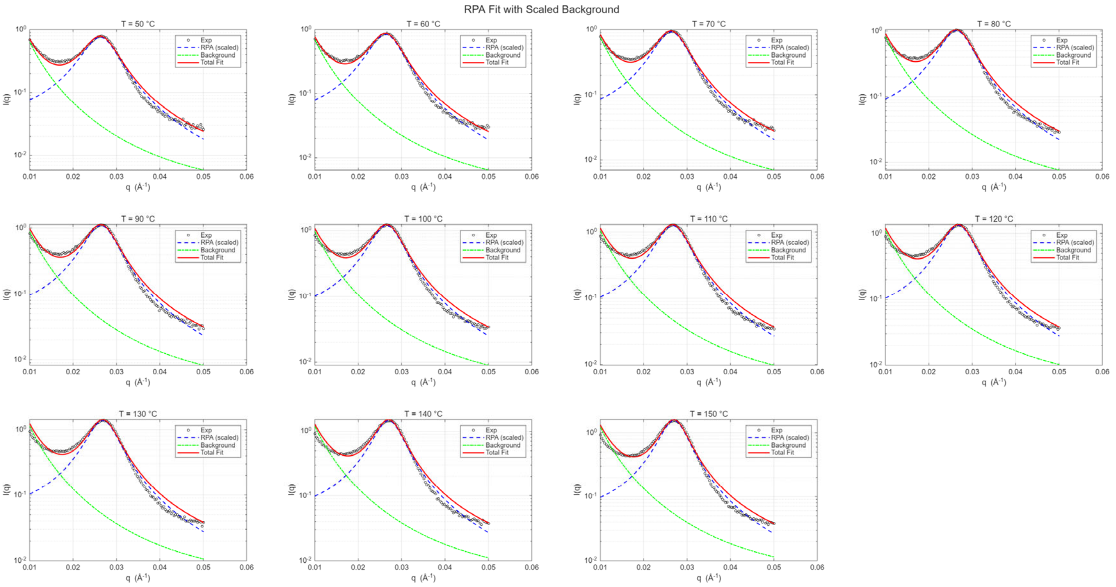

Figure S34. RPA fitting results of PDMS5.8kPS12.5K at different temperatures.

Gaussian Peak Fitting Result: Porod Invariant

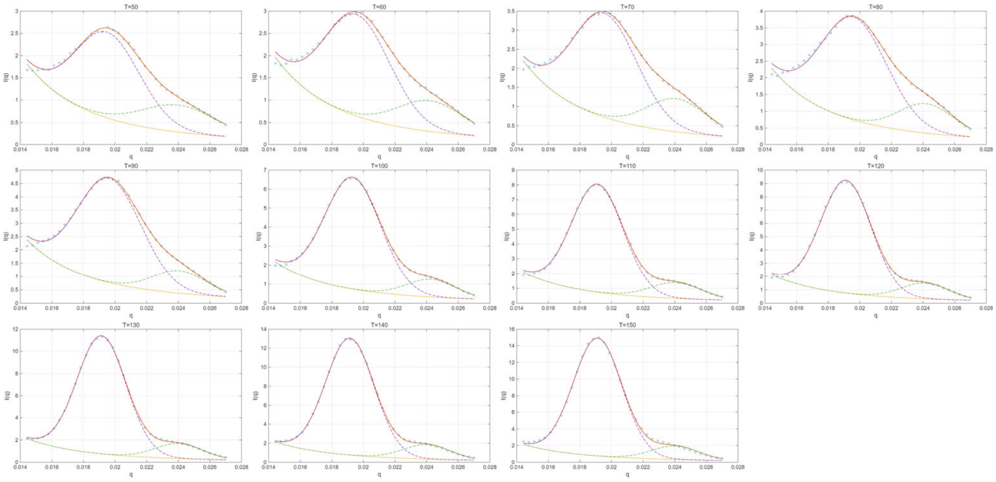

Figure S35. PDMS10.5KPS8.9K r = 1 Gaussian Peaks fitting result of I vs q at different temperature: experimental data(blue), total fit(red), Gaussian Peak 1(purple), Gaussian Peak 2(green), Background(yellow)

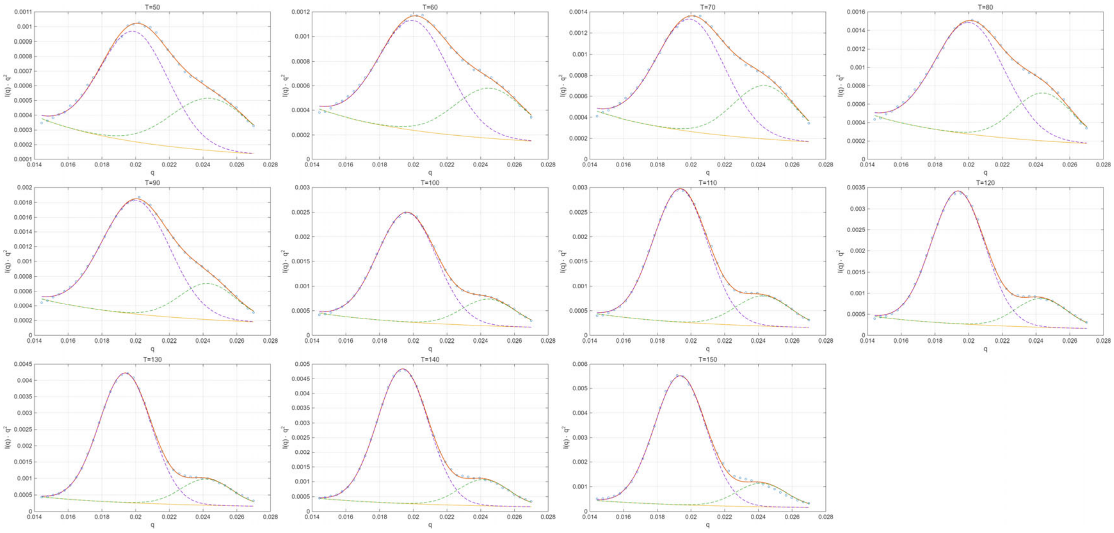

Figure S36. PDMS10.5KPS8.9K r = 1 Gaussian Peaks fitting result of Iq^2 vs q at different temperature: experimental data(blue), total fit(red), Gaussian Peak 1(purple), Gaussian Peak 2(green), Background(yellow)

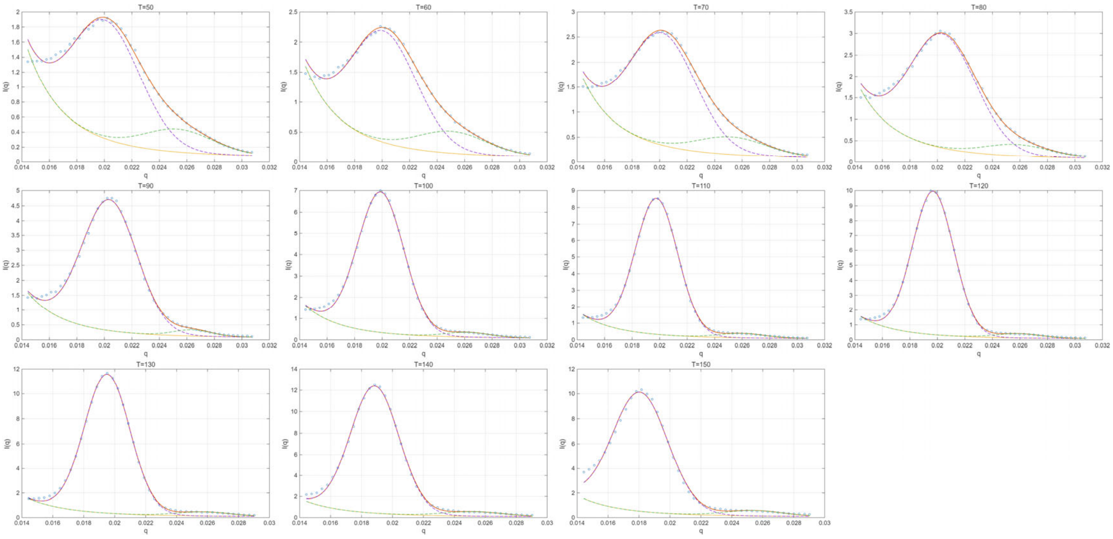

Figure S37. PDMS10.5KPS8.9K r = 1.3 Blend Gaussian Peaks fitting result of I vs q at different temperature: experimental data(blue), total fit(red), Gaussian Peak 1(purple), Gaussian Peak 2(green), Background(yellow)

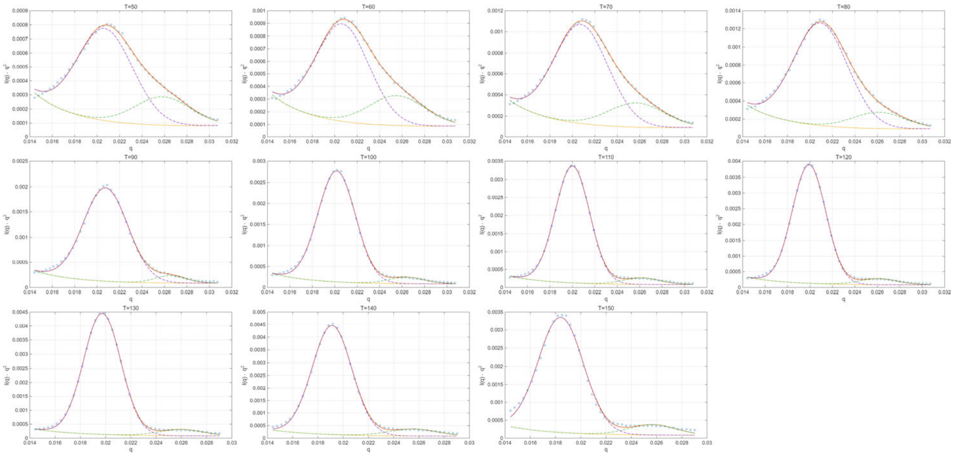

Figure S38. PDMS10.5KPS8.9K r = 1.3 Blend Gaussian Peaks fitting result of Iq^2 vs q at different temperature: experimental data (blue), total fit (red), Gaussian Peak 1 (purple), Gaussian Peak 2 (green), Background (yellow)

Gaussian peak fitting is used to determine the ratio of lamellae to cylinder structures, as shown in the **Figure S35-38**. Similar to the method introduced in RPA fitting, a background function is defined to compensate for errors in background subtraction. First, Gaussian peak fitting is applied to $I(q)$, and then the fitted data is used to generate $Iq^2$and compared Iq^2 from the original data to check the fitting quality. The fitting result is shown in **Figure S35-38**. The integration of the fitting range (Q) is calculated using the fitted Gaussian peaks, see Figure 7 in main text.

PDMS5.8kPS8.9k Blends: Effect of Mixing Ratio

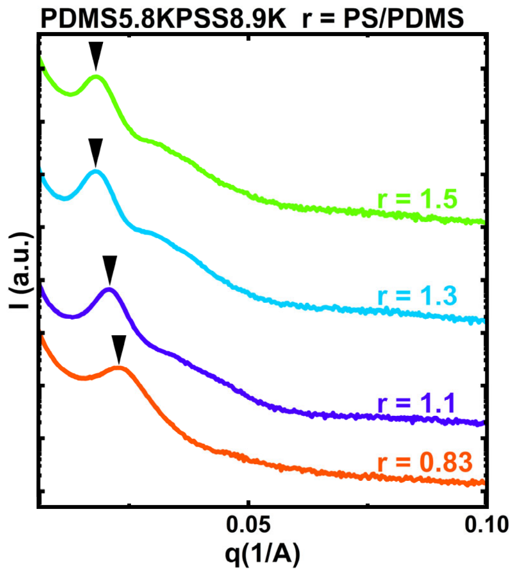

Figure 39. SAXS profiles of PDMS5.8KPS8.9K blends with different mixing ratios at 150 °C.

To further increase the degree of end group association, the mixing ratio of the blends is tuned. Noro previous reported the influence of mixing ratio on the nanostructure of AB telechelic PS/PI blends, where the equimolar blend resulted in a disordered structure, while blends with a molar ratio of 3:1 resulted in a lamellar structure.[51] **Figure 39** shows the SAXS results of PDMS5.8K/PS8.9K blends at different mixing ratios taken at 150 °C. Temperature-dependent SAXS profiles for each blend ratio are provided in the SI, **Figure S21**. While the scattering intensity increases with increasing temperature, the nanoscale structure of the blends is stable with $T_S = 150$ °C for all blends. For the majority PDMS blend, *r* = 0.83, the SAXS profile shows only a broad scattering peak indicative of a disordered structure similar to the equimolar *r* = 1 blend shown in **Figure 3**. However, as the mixing ratio increases from *r* = 1.1 to *r* = 1.3, corresponding to an increase in PS concentration, the primary peak becomes sharper, and a second-order peak emerges, indicating an increase in long-range order. D_0 also increases from 25 nm to 35 nm. We hypothesize that this is due to an increase in the degree of homopolymer association

as the number of PS end groups is increased and the excess PS swell the phase. As *r* is further increased to 1.5, no further changes are observed to the SAXS profile. Due to the presence of higher order peaks, we are unable to apply Tanaka's model to extract quantitative value of *z* for these blends. The SAXS peaks are still broad and the higher order peaks are not sharp enough to indicate the formation of an ordered phase with long-range order. These results agree with findings from Noro[51], who showed that assembled blends can form nanostructures that lack long-range order and exhibit scattering patterns with broad higher order peaks that cannot be assigned to confirm nanoscale morphology. We hypothesize that this is due to an insufficient segregation strength to fully overcome the ODT.

Reversibility of Phase Transitions

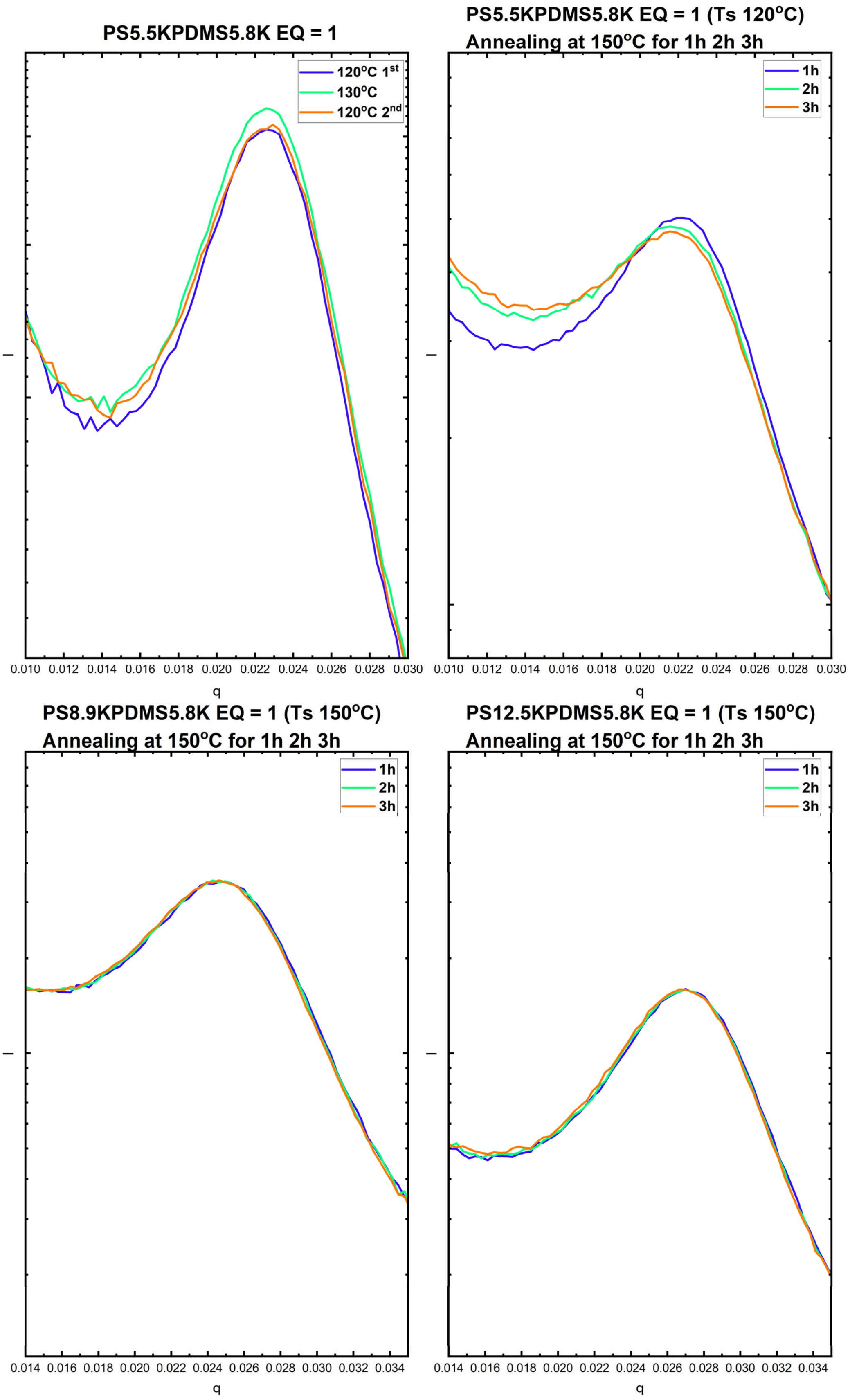

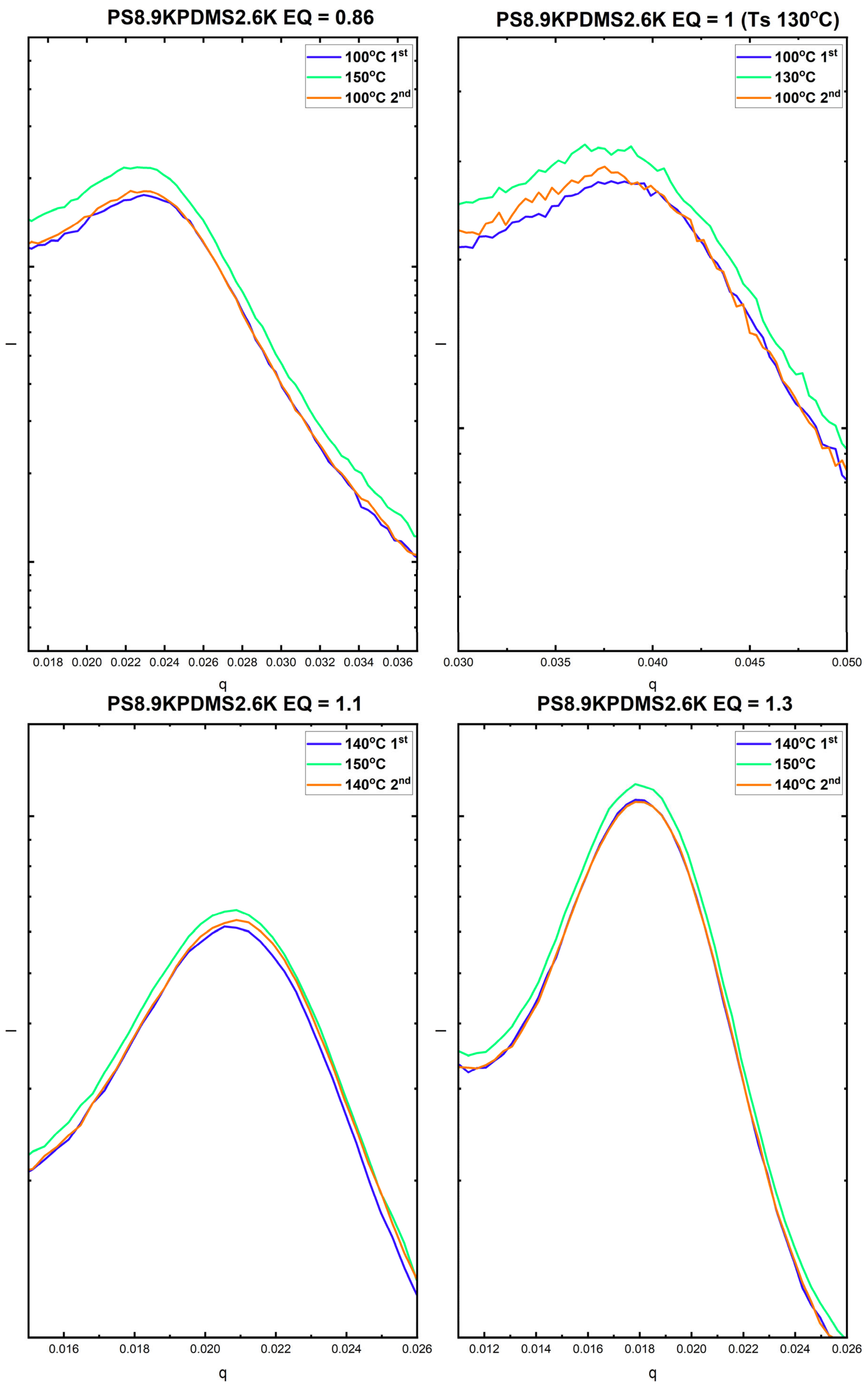
PS8.9KPDMS2.6K EQ = 0.86
100°C 1st
150°C
100°C 2nd
0.018 0.020 0.022 0.024 0.026 0.028 0.030 0.032 0.034 0.036
q
PS8.9KPDMS2.6K EQ = 1 (Ts 130°C)
100°C 1st
130°C
100°C 2nd
0.030 0.035 0.040 0.045 0.050
q
PS8.9KPDMS2.6K EQ = 1.1
140°C 1st
150°C
140°C 2nd
0.016 0.018 0.020 0.022 0.024 0.026
q
PS8.9KPDMS2.6K EQ = 1.3
140°C 1st
150°C
140°C 2nd
0.012 0.014 0.016 0.018 0.020 0.022 0.024 0.026
q

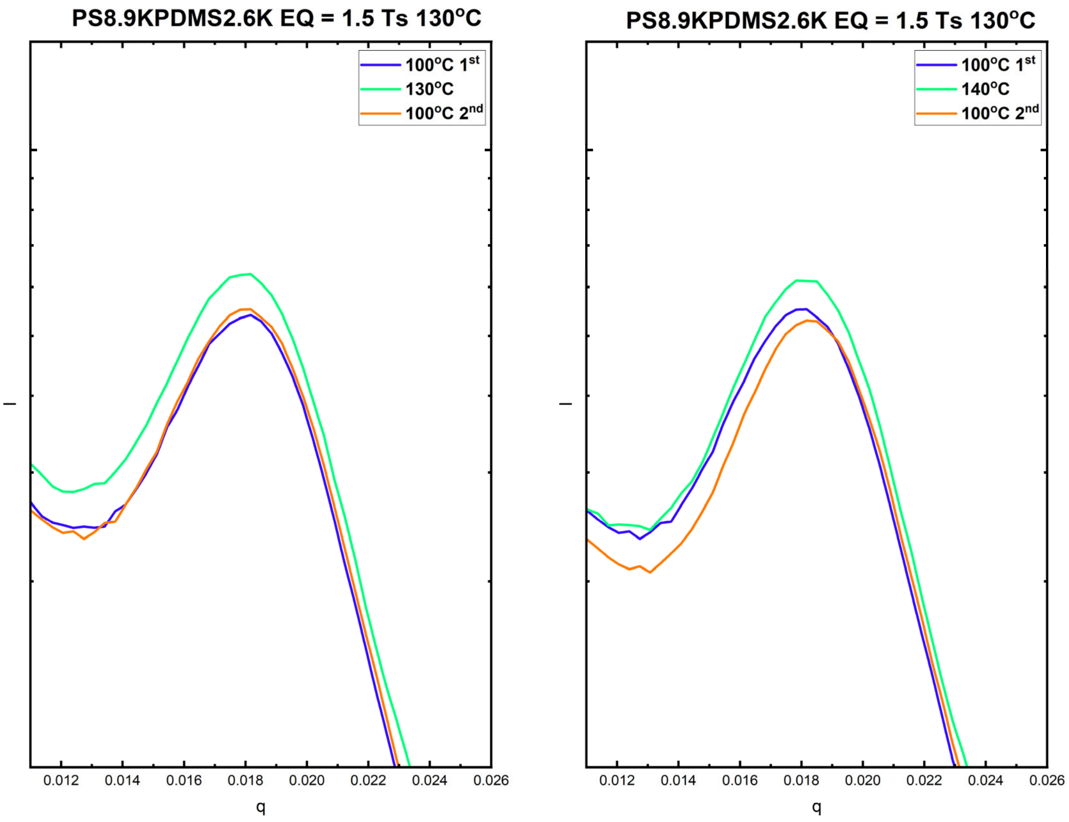

Figure S40. SAXS profiles of different blends under different heating cycles.
